# Extremophile Poeciliidae: multivariate insights into the complexity of speciation along replicated ecological gradients

**DOI:** 10.1186/s12862-016-0705-1

**Published:** 2016-06-22

**Authors:** Rüdiger Riesch, Michael Tobler, Hannes Lerp, Jonas Jourdan, Tess Doumas, Patrik Nosil, R. Brian Langerhans, Martin Plath

**Affiliations:** School of Biological Sciences, Royal Holloway University of London, Egham, Surrey TW20 0EX UK; Department of Biological Sciences & W. M. Keck Center for Behavioral Biology, North Carolina State University, 127 David Clark Labs, Raleigh, NC 27695-7617 USA; Division of Biology, Kansas State University, 116 Ackert Hall, Manhattan, KS 66506 USA; Natural History Collections, Museum Wiesbaden, Friedrich-Ebert-Allee 2, 65185 Wiesbaden, Germany; J. W. Goethe-University Frankfurt/M., Evolutionary Ecology Group, Max-von-Laue Str. 13, 60438 Frankfurt a. M., Germany; Department of Biology and Biochemistry, University of Houston, 4800 Calhoun Rd., Houston, TX 77004 USA; Department of Animal and Plant Sciences, University of Sheffield, Sheffield, S10 2TN UK; College of Animal Science and Technology, Northwest A&F University, Xinong Road 22, Yangling, 712100 People’s Republic of China

**Keywords:** Hydrogen sulfide, *Gambusia*, Ecological speciation, Life-history evolution, Morphometrics, *Poecilia*, Reproductive isolation

## Abstract

**Background:**

Replicate population pairs that diverge in response to similar selective regimes allow for an investigation of (*a*) whether phenotypic traits diverge in a similar and predictable fashion, (*b*) whether there is gradual variation in phenotypic divergence reflecting variation in the strength of natural selection among populations, (*c*) whether the extent of this divergence is correlated between multiple character suites (i.e., concerted evolution), and (*d*) whether gradual variation in phenotypic divergence predicts the degree of reproductive isolation, pointing towards a role for adaptation as a driver of (ecological) speciation. Here, we use poeciliid fishes of the genera *Gambusia* and *Poecilia* that have repeatedly evolved extremophile lineages able to tolerate high and sustained levels of toxic hydrogen sulfide (H_2_S) to answer these questions.

**Results:**

We investigated evolutionary divergence in response to H_2_S in *Gambusia* spp. (and to a lesser extent *Poecilia* spp.) using a multivariate approach considering the interplay of life history, body shape, and population genetics (nuclear miscrosatellites to infer population genetic differentiation as a proxy for reproductive isolation). We uncovered both shared and unique patterns of evolution: most extremophile *Gambusia* predictably evolved larger heads and offspring size, matching a priori predictions for adaptation to sulfidic waters, while variation in adult life histories was idiosyncratic. When investigating patterns for both genera (*Gambusia* and *Poecilia*), we found that divergence in offspring-related life histories and body shape were positively correlated across populations, but evidence for individual-level associations between the two character suites was limited, suggesting that genetic linkage, developmental interdependencies, or pleiotropic effects do not explain patterns of concerted evolution. We further found that phenotypic divergence was positively correlated with both environmental H_2_S-concentration and neutral genetic differentiation (a proxy for gene flow).

**Conclusions:**

Our results suggest that higher toxicity exerts stronger selection, and that divergent selection appears to constrain gene flow, supporting a scenario of ecological speciation. Nonetheless, progress toward ecological speciation was variable, partially reflecting variation in the strength of divergent selection, highlighting the complexity of selective regimes even in natural systems that are seemingly governed by a single, strong selective agent.

**Electronic supplementary material:**

The online version of this article (doi:10.1186/s12862-016-0705-1) contains supplementary material, which is available to authorized users.

## Background

Darwin’s [1] view that speciation can be thought of as a continuum has gained increasing traction in recent years (e.g., [2–10]). Within this conceptual framework, pairs of populations move bidirectionally along a continuum between panmixis and complete reproductive isolation: populations can either progress towards speciation or collapse back into panmixis even after substantial population divergence (e.g., [11–15]). Several factors shape the speciation continuum and determine where a given population pair falls along it. All else equal (i.e., assuming that the same speciation mechanisms act in replicate population pairs), progress towards complete speciation should increase as a function of time, with populations undergoing divergent selection or genetic drift for longer time periods moving farther along the continuum toward total reproductive isolation than populations that have diverged more recently. However, ‘all else’ is usually not equal, and so differences in progress towards complete speciation can also be explained by additional or stronger selective agents at play in only some replicate populations, mutation-order effects, differences in the genetic architecture of the founder populations, and/or stochastic effects (e.g., [7, 8, 16]).

**Fig. 1:**
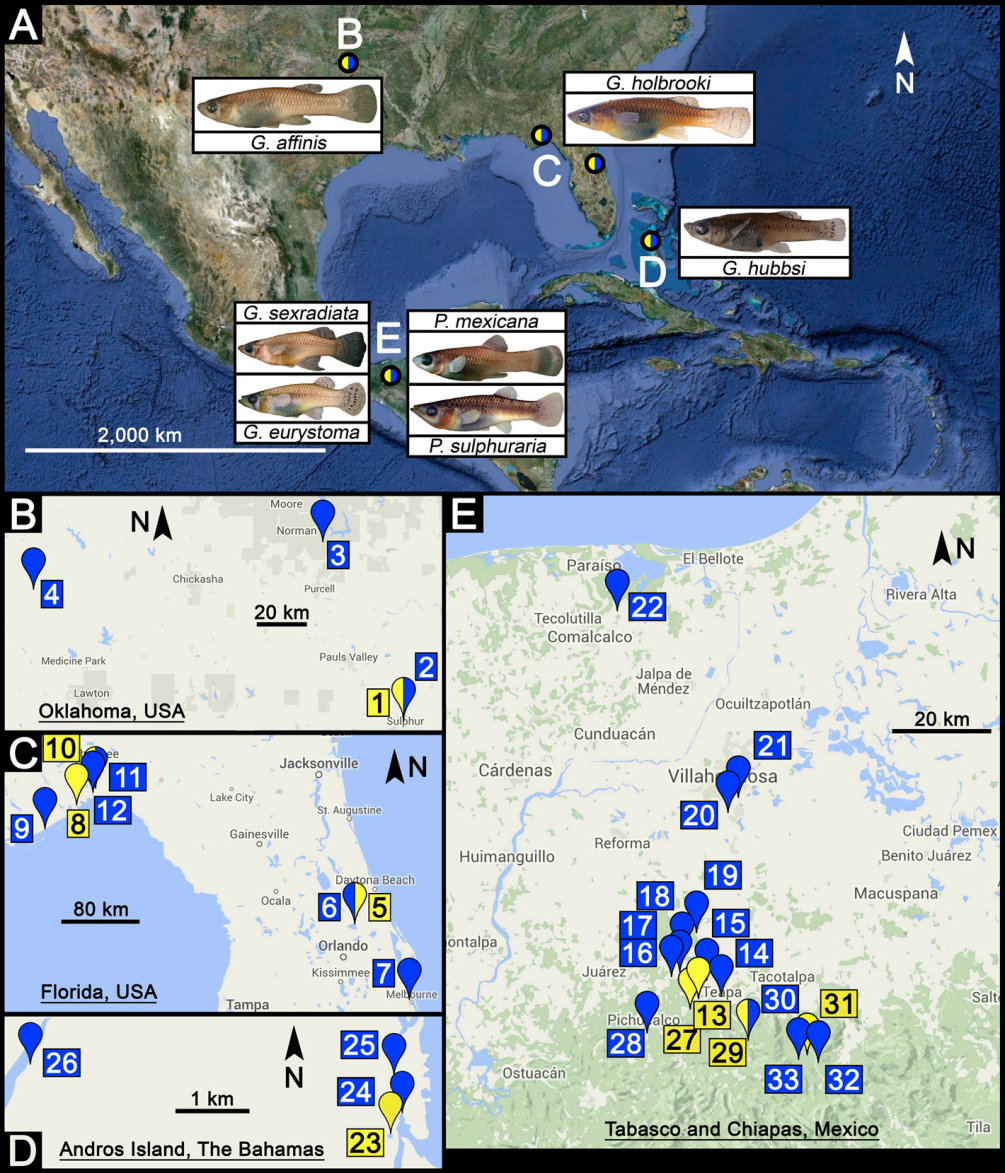
**a** Overview of the general study area. (B-E) Detailed views of collection sites for (**b**) *Gambusia affinis* in Oklahoma, (**c**) *Gambusia holbrooki* in Florida, (**d**) *Gambusia hubbsi* from northern Andros Island, The Bahamas, and (**e**) *Gambusia eurystoma* and *Gambusia sexradiata* in Tabasco, southern Mexico. Numbers correspond to sampling sites as described in Table 1. Yellow: sulfidic habitats; blue: non-sulfidic sites. Panel (**a**) was created using GOOGLE EARTH VS. 7.1.5.1557 (©2015 Google Inc., Mountain View, CA, USA), while panels (**b**–**e**) were created using GOOGLE MAPS (Map data ©2015 Google, INEGI)

The comparative approach, using multiple populations across replicated environmental gradients, not only provides a tool for uncovering mechanisms underlying speciation in natural systems—i.e., how reproductive isolation arises—but also patterns of convergent and non-convergent trait evolution (e.g., [7, 8, 17–19]). Nonetheless, even comparative studies usually do not trace progression toward speciation in a single lineage through time but rather use comparisons among closely related taxa that have reached different stages of divergence, the underlying assumption being that a single lineage would follow a similar course through time as speciation unfolds (see also discussion in [20]). Studies employing such a comparative approach have usually focused on single traits or character suites, exemplified by several studies on variation in only life history or morphology (e.g., [21–24]). This neglects the possibility that multiple character suites may evolve in concert (e.g., [25–27]). In fact, only by simultaneously investigating multiple types of traits in conjunction with pertinent environmental features can we more fully understand the complexity of adaptive diversification and how speciation unfolds in natural systems [7, 8, 25, 27–29]. In our present study, we use livebearing fishes (family Poeciliidae) of the genera *Gambusia* (mosquitofishes) and *Poecilia* (mollies) to assess divergence in multiple phenotypic character suites (i.e., life history and morphology) across a geographically replicated ecological gradient created by toxic hydrogen sulfide (H_2_S) (Fig. 1 and Table 1). We further measure the degree of neutral genetic differentiation between geographically adjacent population pairs as a proxy for gene flow—and thus, as an estimate of where population pairs reside along the speciation continuum.

**Table 1:** Overview of sample sites and sample sizes used for analyses

ID	Clade	Site	H_2_S	Latitude	Longitude	Life History	Morphology	Pop. Gen.
(1)	*Gambusia affinis*	Vendome Well	+	34°30'21.28"N	96°58'19.51"W	46 ♂, 31♀	27♂, 31♀^(1)^	–
(2)		Travertine Creek	–	34°30'14.90"N	96°58'16.68"W	11♂, 44♀	10♂, 31♀^(1)^	–
(3)		Dave Blue Creek	–	35°11'21.12"N	97°20'48.84"W	–	24♂, 22♀^(1)^	–
(4)		Trib. Washita River	–	35°0'16.38"N	98°41'21.49"W	21♂, 29♀	–	–
(5)	*Gambusia holbrooki*	Green Springs	+	28°51'47.06"N	81°14'54.83"W	23♂, 33♀	23♂, 58♀	22
(6)		Lake Monroe	–	28°8'40.03"N	80°35'50.39"W	20♂, 29♀	20♂, 37♀	22
(7)		Flood Drain	–	28°7'43.08"N	80°38'6.66"W	–	–	23
(8)		Panacea Mineral Springs	+	30°2'4.14"N	84°23'23.34"W	13♂, 26♀	13♂, 35♀	17
(9)		Ditch Hwy 98	–	29°47'52.32"N	84°44'40.68"W	18♂, 31♀	18♂, 35♀	22
(10)		Newport Springs	+	28°51'44.39"N	81°15'9.91"W	24♂, 44♀	24♂, 49♀	20
(11)		St. Marks River	–	30°11'57.33"N	84°10'40.00"W	–	–	24
(12)		St. Marks Ditch	–	30°9'16.46"N	84°12'19.52"W	24♂, 43♀	24♂, 44♀	24
(13)	*Gambusia eurystoma*	Baños del Azufre	+	17°33'9.20"N	92°59'51.45"W	27♂, 14♀	35♂, 64♀	24
(14)	*Gambusia sexradiata*	Río Teapao	–	17°33'24.90"N	92°57'3.54"W	22♂, 30♀	–	–
(15)		Laguna Canto Rodado	–	17°35'20.40"N	92°58'51.60"W	–	6♂, 24♀	–
(16)		Pichucalco (1)	–	17°35'43.40"N	93°3'12.80"W	–	6♂, 32♀	–
(17)		Pichucalco (2)	–	17°36'18.94"N	93°2'10.31"W	–	16♂, 31♀	24
(18)		Pichucalco (3)	–	17°38'26.60"N	93°1'53.33"W	–	–	20
(19)		Laguna West	–	17°40'55.22"N	93°0'8.13"W	–	–	19
(20)		Parrilla/Lima	–	17°54'59.85"N	92°56'15.37"W	–	–	24
(21)		Café Villahermosa	–	17°56'46.14"N	92°54'57.13"W	–	–	24
(22)		Ditch near Chichicapa	–	18°18'39.75"N	93° 9'54.21"W	–	4♂, 31♀	–
(23)	*Gambusia hubbsi*	Archie’s blue hole	+	24°54'4.56"N	77°56'10.77"W	20♂, 36♀	21♂, 45♀	24
(24)		Stafford Creek North	–	24°54'14.95"N	77°56'5.00"W	–	–	24
(25)		London Pond	–	24°54'35.99"N	77°59'4.00"W	15♂, 26♀	8♂, 32♀	–
(26)		Thompson/Scott	–	24°54'32.03"N	77°56'8.99"W	19♂, 11♀	9♂, 33♀	25
(13)	*Poecilia sulphuraria*	Baños del Azufre	+	17°33'9.20"N	92°59'51.45"W	26♀^(2)^	89♂, 118♀^(3)^	25^(4)^
(27)		La Gloria	+	17°31'55.24"N	93° 0'54.47"W	27♀	29♂, 28♀^(3)^	24^(4)^
(28)	*Poecilia mexicana*	Arroyo Rosita	–	17°29'6.40"N	93° 6'12.89"W	37♀	29♂, 27♀^(3)^	25^(4)^
(29)		La Lluvia	+	17°27'49.99"N	92°53'43.48"W	15♀	25♂, 40♀^(3)^	25^(5)^
(30)		Río Puyacatengo	–	17°28'12.00"N	92°53'44.63"W	31♀	45♂, 31♀^(3)^	25^(5)^
(31)		El Azufre	+	17°26'32.10"N	92°46'28.09"W	23♀^(2)^	47♂, 47♀^(3)^	25^(5)^
(32)		Arroyo Bonita	–	17°25'37.42"N	92°45'6.98"W	33♀^(2)^	24♂, 14♀^(3)^	25^(5)^
(33)		Río Amatan	–	17°25'59.92"N	92°47'34.55"W	23♀^(2)^	30♂, 15♀^(3)^	-

^(1)^ reanalyzed from [47]; ^(2)^ reanalyzed from [24]; ^(3)^ reanalyzed from [40]; ^(4)^ reanalyzed from [48]; ^(5)^ reanalyzed from [9]

To make specific predictions about the evolution of life histories and morphology, we need a clear understanding of the various sources of selection in sulfidic habitats. H_2_S toxicity results predominantly from its interference with mitochondrial bioenergetics, which inhibits oxidative phosphorylation [30, 31]. The suppression of aerobic respiration is aggravated by the reactivity of H_2_S leading to extreme hypoxia in aquatic environments; nevertheless, a number of livebearing fishes (Poeciliidae) have successfully colonized naturally sulfidic habitats [31]. Adaptation to H_2_S has been studied especially within the genus *Poecilia* from southern Mexico, where evolutionarily independent lineages have colonized sulfidic springs in four river drainages (e.g., [9, 32–40]). Living in H_2_S-rich environments should be accompanied by life-history trait modifications, particularly through selection on offspring size [41]. First, as the ratio of sulfide influx to sulfide oxidation is crucial for efficient detoxification, larger neonates are expected to exhibit higher tolerance to elevated environmental H_2_S concentrations due to a lower body surface-to-volume ratio [42, 43]. Second, larger neonates are expected to have a competitive advantage in sulfidic habitats, which are thought to be resource-limited [31, 44, 45]. Congruent with these considerations, extremophile poeciliids of nine different species are characterized by larger offspring size at birth, and as a result of the classic life-history trade-off between offspring size and fecundity [46], are further characterized by a reduced fecundity compared to their closest relatives from non-sulfidic waters [41]. The question remains, however, whether larger offspring size in extremophile poeciliids is indeed a ubiquitous response to exposure to even low concentrations of environmental H_2_S, or whether extremophile populations could be found that lack this adaptation.

With respect to morphology, divergence in southern Mexico is mainly the result of extremophile *Poecilia* having larger heads coupled with an increased gill surface area to enhance respiratory capacities—and thus survival—in hypoxic H_2_S-springs [32, 34, 47]. Besides its direct impact on survival, relative head size may also provide a cue mediating mate choice, as female *Poecilia* in southern Mexican streams prefer males from their own population over immigrant (sulfide-adapted) males [9, 33].

Here, we start by focusing on all mosquitofish populations known to inhabit sulfidic waters. Specifically, we sampled four different clades of mosquitofish (*G. affinis*, *G. holbrooki*, *G. hubbsi*, and the two sister species *G. sexradiata* and *G. eurystoma*) to address the question of how predictable and convergent phenotypic divergence is in response to H_2_S among *Gambusia* species (question 1). Based on physiological studies, life-history theory, and previous work on extremophile *Poecilia* spp. (see above), we predicted extremophile mosquitofish to have larger but fewer offspring, and to develop larger heads compared to their closest relatives from non-sulfidic waters. We also examined other traits (e.g., adult fat content, reproductive allocation, overall body shape) that might diverge as a result of resource limitation (e.g., less body fat in sulfidic habitats) and higher population densities (e.g., more streamlined body shapes in sulfidic habitats), but for which previous studies on extremophile poeciliids from southern Mexico did not always find convergent and predictable patterns [24].

We then integrated the mosquitofish data with data collected on extremophile *Poecilia* spp. [9, 24, 40, 47, 48] to address two additional questions: First, is the strength of any observed shift in one of the character suites (life history or morphology) correlated with divergence in the other (question 2)? If developmental interdependencies, genetic linkage, or pleiotropy play important roles for phenotypic diversification in extremophile *Gambusia*, we would expect correlations between these character suites both within (i.e., on the inter-individual level) and between populations. Alternatively, the presence of H_2_S could create multifarious selective regimes that act on suites of traits not necessarily developmentally or genetically integrated. In that case we would predict trait correlations between body shape variation and important life-history traits (e.g., offspring size and fecundity) between, but not within, populations.

Moreover, we asked whether divergent selection between toxic and benign environments generally results in reduced gene flow (estimated through a population genetic approach using nuclear microsatellites), and if stronger phenotypic divergence is typically correlated with greater reductions in gene flow (question 3)? For the first time, we thus approach ecological speciation in H_2_S-rich environments from a multivariate perspective that considers the interplay of life history, morphology, and population genetics. We focused on both shared and unique patterns of population divergence (or the lack thereof) and provide a unifying framework within which variable progress along the speciation continuum can be interpreted.

## Results

### Life-history divergence

#### Adult life histories

In the ANOVA on SL using the subset of data from *Gambusia* spp., the factors sex, clade, and site(clade × H_2_S), as well as the interactions of sex × clade, sex × H_2_S, and sex × clade × H_2_S had a significant influence, while the presence of H_2_S itself was not significant (Table 2a). Based on *η*_p_^2^, the most important predictors for size differences were sex, clade and site(clade × H_2_S) (Table 2a). Males were generally smaller than females (estimated marginal means, EMM ± s.e., males: 20.85 ± 0.17 mm; females: 27.49 ± 0.15 mm; sex-effect in Table 2a); however, clades also differed in size (clade-effect) and within each clade, populations from the same habitat type also differed strongly from each other (effect of site(clade × H_2_S)).

**Table 2:** Results of nested univariate analysis of variance (ANOVA) and multivariate analyses of covariance (MANCOVA) examining life-history and body shape variation of *Gambusia* spp. from four different clades, comparing sulfidic springs versus non-sulfidic habitats. The site(clade × H_2_S)-term was designated a random effect in all models. *F*-ratios in the MANCOVAs were approximated using Wilks’ values, partial variance was estimated using Wilks’ partial *η* ^2^, and terms with a partial variance at least half as strong as the strongest effect are given in bold for each model. Asterisks indicate terms that were tested using the mixed-model approach (see text)

Effect	*F*	df	*P*	Partial variance	Relative variance
*(a) SL (ANOVA)*					
**Sex**	**817.5**	**1, 708**	**< 0.001**	**0.127**	**0.516**
**Clade**	**5.0**	**3, 6**	**0.044**	**0.246**	**1.000**
H_2_S	2.7	1, 6	0.15	0.052	0.211
**Site(Clade × H**_**2**_ **S)**	**17.2**	**6, 708**	**< 0.001**	**0.127**	**0.516**
Sex **×** Clade	5.8	3, 708	0.001	0.024	0.098
Sex **×** H_2_S	8.5	1, 708	0.004	0.012	0.049
Sex **×** Clade **×** H_2_S	30.5	3, 708	< 0.001	0.114	0.463
*(b) Adult life histories (MANCOVA)*					
**SL**	**2211.5**	**3, 702**	**< 0.001**	**0.904**	**1.000**
**Sex**	**513.3**	**3, 702**	**< 0.001**	**0.687**	**0.760**
Clade*	41.3	6, 1342	< 0.001	0.147	0.163
H_2_S*	3.4	2, 1121	0.0342	0.161	0.178
Site(Clade **×** H_2_S)	17.8	18, 1986	< 0.001	0.132	0.146
Clade **×** H_2_S*	8.5	6, 1342	< 0.001	0.096	0.106
SL **×** Clade	1.5	9, 1708.6	0.007	0.011	0.012
Sex **×** Clade	4.1	9, 1708.6	< 0.001	0.017	0.019
Sex **×** H_2_S	15.5	3, 702	< 0.001	0.062	0.069
Sex **×** Clade **×** H_2_S	7.3	9, 1708.6	< 0.001	0.030	0.033
*(c) Offspring-related life histories (MANCOVA)*					
**SL**	**69.9**	**3, 395**	**< 0.001**	**0.347**	**0.569**
Stage of Development	16.0	3, 395	< 0.001	0.108	0.177
Clade*	22.3	6, 934	< 0.001	0.297	0.488
**H**_**2**_ **S**	**54.6**	**2, 771**	**< 0.001**	**0.609**	**1.000**
Site(Clade **×** H_2_S)	24.9	18, 1117.7	< 0.001	0.272	0.447
Clade **×** H_2_S*	5.2	6, 934	< 0.001	0.124	0.203
SL **×** Clade	5.4	9, 961.5	< 0.001	0.039	0.065
SL **×** H_2_S	3.4	3, 395	0.019	0.025	0.041
SL **×** Clade **×** H_2_S	2.0	9, 961.5	0.038	0.015	0.024
Stage **×** Clade	3.4	9, 961.5	0.0004	0.025	0.041
Stage **×** H_2_S	2.9	3, 395	0.034	0.022	0.036
Stage **×** Clade **×** H_2_S	2.4	9, 961.5	0.011	0.018	0.029
*(d) Body shape (MANCOVA)*					
**Centroid size**	**125.0**	**7, 898**	**< 0.001**	**0.512**	**0.651**
**Sex**	**129.8**	**7, 898**	**< 0.001**	**0.504**	**0.640**
Clade*	**226.8**	**18, 4227**	**< 0.001**	**0.787**	**1.000**
**H**_**2**_ S*	**22.3**	**6, 2809**	**< 0.001**	**0.403**	**0.513**
Site(Clade **×** H_2_S)	13.2	63, 5063.7	< 0.001	0.160	0.204
Clade **×** H_2_S*	19.4	18, 4227	< 0.001	0.297	0.378

**Fig. 2:**
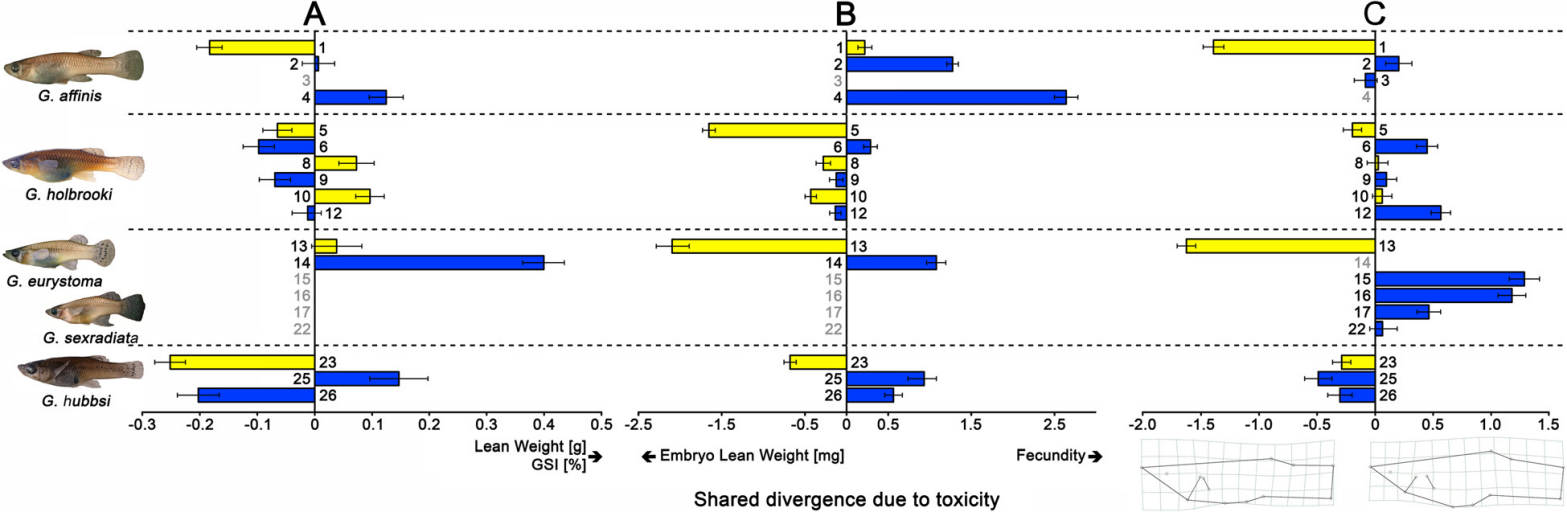
**a** Adult life-history, (**b**) offspring-related life-history, and (**c**) body shape divergence between *Gambusia* spp. populations from sulfidic habitats (*yellow*) and non-sulfidic sites (*blue*). Depicted are mean divergence vector scores (± s.e.; derived from the H_2_S term in the MANCOVAs) for each site; these describe the linear combination of dependent variables exhibiting the greatest difference between sulfidic and non-sulfidic habitats in Euclidean space (please see Additional file 1: OSM 3 for details). Numbers correspond to sites as described in Table 1

In the mixed-model nested MANCOVA on adult life histories, the covariate SL, the factors sex, H_2_S, clade, and site(clade × H_2_S), as well as the interactions of SL × clade, sex × clade, sex × H_2_S, clade × H_2_S, and sex × clade × H_2_S had a significant influence on the examined traits (Table 2b). Based on our measure of effect size (*η*_p_^2^), SL and sex had by far the strongest influence on adult life histories while the factor H_2_S was of minor importance, and no consistent pattern of divergence due to the presence of H_2_S was uncovered (Table 2b; Fig. 2a). All three life-history traits generally increased with increasing SL (Pearson correlation, lean weight: *r*_p_ = 0.94, *P* < 0.0001; fat content: *r*_p_ = 0.18, *P* < 0.0001; GSI: *r*_p_ = 0.65, *P* < 0.0001). Compared to similar-sized females, males weighed more (EMM ± s.e. at SL = 25.19 mm, lean weight, males: 0.083 ± 0.001 g, females: 0.067 ± 0.001 g), but had less body fat (males: 5.23 ± 0.44 %, females: 6.45 ± 0.32 %), and had a smaller relative investment into reproduction (GSI, males: 1.68 ± 0.37 %, RA, females: 17.70 ± 0.27 %; Table 2b and Additional file 1: Table S3). Lean weight, fat content, and GSI responded inconsistently to the presence of H_2_S across clades and sexes (Additional file 1: Table S3).

Descriptive statistics (site-specific means) for all *N* = 730 individuals analyzed for life history traits are summarized in Additional file 1: Tables S1 and S2.

#### Offspring-related life histories

In the mixed-model nested MANCOVA on offspring-related life histories, the covariates SL and stage of development, the factors H_2_S, clade, and site(clade × H_2_S), as well as the interactions clade × H_2_S, SL × clade, SL × H_2_S, stage of development × clade, and stage of development × H_2_S had a significant influence on the evaluated traits (Table 2c); however, based on *η*_p_^2^, the presence of H_2_S and SL had by far the strongest influence (Table 2c). As predicted, the H_2_S-effect was mainly due to differences in embryo lean weight and fecundity (Fig. 2b), with females in sulfidic habitats producing significantly larger but fewer offspring (EMM ± s.e. for SL = 28.07 mm and stage of development = 27.87, embryo lean weight: non-sulfidic = 1.52 ± 0.04 mg, sulfidic = 3.04 ± 0.03 mg; fecundity: non-sulfidic = 14.98 ± 0.75 mg, sulfidic = 9.09 ± 0.66 mg; Additional file 1: Table S3). Fecundity increased with increasing female SL, but embryo fat content and embryo lean weight did not vary with female size (fecundity: *r*_p_ = +0.67, *P* < 0.0001; embryo fat content: *r*_p_ = -0.06, *P* = 0.24; embryo lean weight: *r*_p_ = -0.08, *P* = 0.10).

### Morphological divergence

In total, we examined *N* = 914 individuals of *Gambusia* spp. from 17 sites (Table 1). Centroid size, sex, clade, H_2_S, site(clade × H_2_S), and the interaction of clade × H_2_S had a significant influence on body shape (Table 2d). According to *η*_p_^2^, the strongest differences were uncovered between clades and sexes, but body shape also differed between fish from sulfidic and non-sulfidic habitats (Table 2d). Thin-plate spline transformation grids visualizing the shape differences along the divergence vector derived from the H_2_S-term indicate that extremophile mosquitofish tended to have larger heads, more slender bodies, elongated caudal peduncles, and more posteriorly positioned dorsal fins (Fig. 2c). The degree of population differentiation varied among clades, with the strongest divergence found in *G. affinis* and the *G. eurystoma/sexradiata* complex (clade-effect), while *G. hubbsi* exhibited a trend toward slightly smaller heads and deeper bodies in the sulfidic habitats (clade × H_2_S-effect; Fig. 2c). The primary difference between sexes was in the position of the anal fin (sex-effect), which is modified into a copulatory organ (gonopodium) in males and positioned more anteriorly than the female anal fin [49].

### Population genetic differentiation

**Fig. 3:**
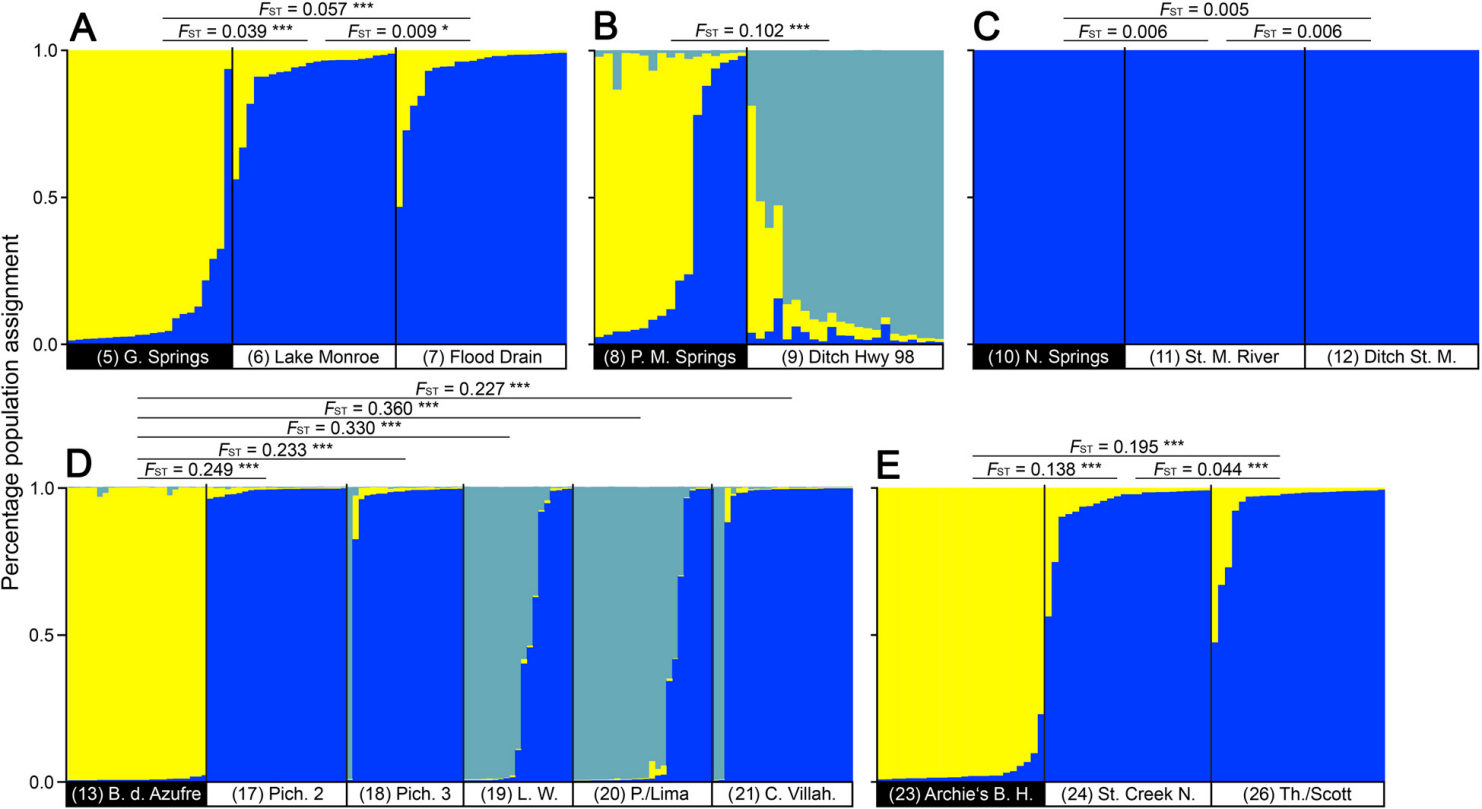
Population assignment using STRUCTURE version 2.3.2. Sulfidic and surrounding non-sulfidic habitats of (**a**–**c**) *G. holbrooki*, (**d**) *G. eurystoma/sexradiata*, and (**e**) *G. hubbsi. K* = 1 was recovered as the most likely number of genetic clusters in **c**, *K* = 2 in **a** and **e**, and *K* = 3 in **b** and **d**; given are individual relative assignment values in yellow (sulfidic habitats; left side) as well as blue and olive (non-sulfidic sites; right side), divided by a black line and sorted by the relative assignment score for each population separately. Numbers correspond to sites as described in Table 1

Descriptive statistics for site-specific means of genetic variability of the *N* = 383 genotyped individuals are provided in Additional file 1: Table S5. We found variable degrees of genetic differentiation between fish from sulfidic and non-sulfidic habitats in *G. holbrooki*, ranging from virtual panmixis (*F*_ST_ = 0.006) to moderate genetic structure (*F*_ST_ = 0.249; Fig. 3a-c). In the Green Springs system, we detected *K* = 2 as the uppermost hierarchical level of population structure, with individuals from population 5 (Green Springs) being assigned to one genetic cluster and individuals from both non-sulfidic populations 6 and 7 (Lake Monroe and Flood Drain) being assigned to another cluster (Fig. 3a). However, the situation in the Florida Panhandle is considerably different. Using all sampling sites in one analysis we detected *K* = 2 clusters with animals originating from population 9 (Ditch Highway 98) being assigned to one and all other individuals (regardless of the presence of H_2_S) being assigned to the other cluster (Additional file 1: Figure S2). To infer the effect of H_2_S on gene flow between adjacent habitats, we decided to split our Florida Panhandle data and conducted separate, independent analyses. For the westernmost cluster of sites, populations 8 and 9 (Panacea Mineral Springs and Ditch Highway), we detected *K* = 3 to be the most likely number of clusters, involving multiple clusters within one site (Fig. 3b). A test for migration or hybridization within the last two generations was conducted using the USEPOPINFO model implemented in STRUCTURE, but could only detect minor probability for recent migration events between both populations (*Q* < 0.09). For the cluster of populations 10 to 12 (Newport Springs, St. Marks River, and Ditch St. Marks), the most likely level of population structure was obtained for *K* = 1, thus pointing towards unimpeded gene flow between sulfidic and non-sulfidic sites (Fig. 3c).

*K* = 3 was uncovered as the uppermost hierarchical level of population structure in Mexico. The sulfidic site inhabited by *G. eurystoma* (population 13) formed a genetic cluster distinct from all *G. sexradiata* populations (populations 17–21; Fig. 3d). Pairwise *F*_ST_-values supported the results obtained from STRUCTURE, being highest in all pairwise comparisons of *G. eurystoma* and *G. sexradiata* (*F*_ST_ ≥ 0.227).

The separation between sulfidic and non-sulfidic sites was also clear for the Bahamas sites, where *K* = 2 was uncovered as the uppermost hierarchical level of population structure: *G. hubbsi* from sulfidic Archie’s Blue Hole (population 23) formed one genetic cluster that was distinct from the nearby non-sulfidic habitats (populations 24 and 26; Fig. 3e).

The partial Mantel test on the global dataset uncovered a trend (albeit non-significant) for stronger differentiation between divergent habitats than between similar habitats (*r*_P_ = 0.11, one-tailed *P* = 0.067), while controlling for differences among clades.

### Joint evolution of life histories, morphologies, and reproductive isolation?

**Fig. 4:**
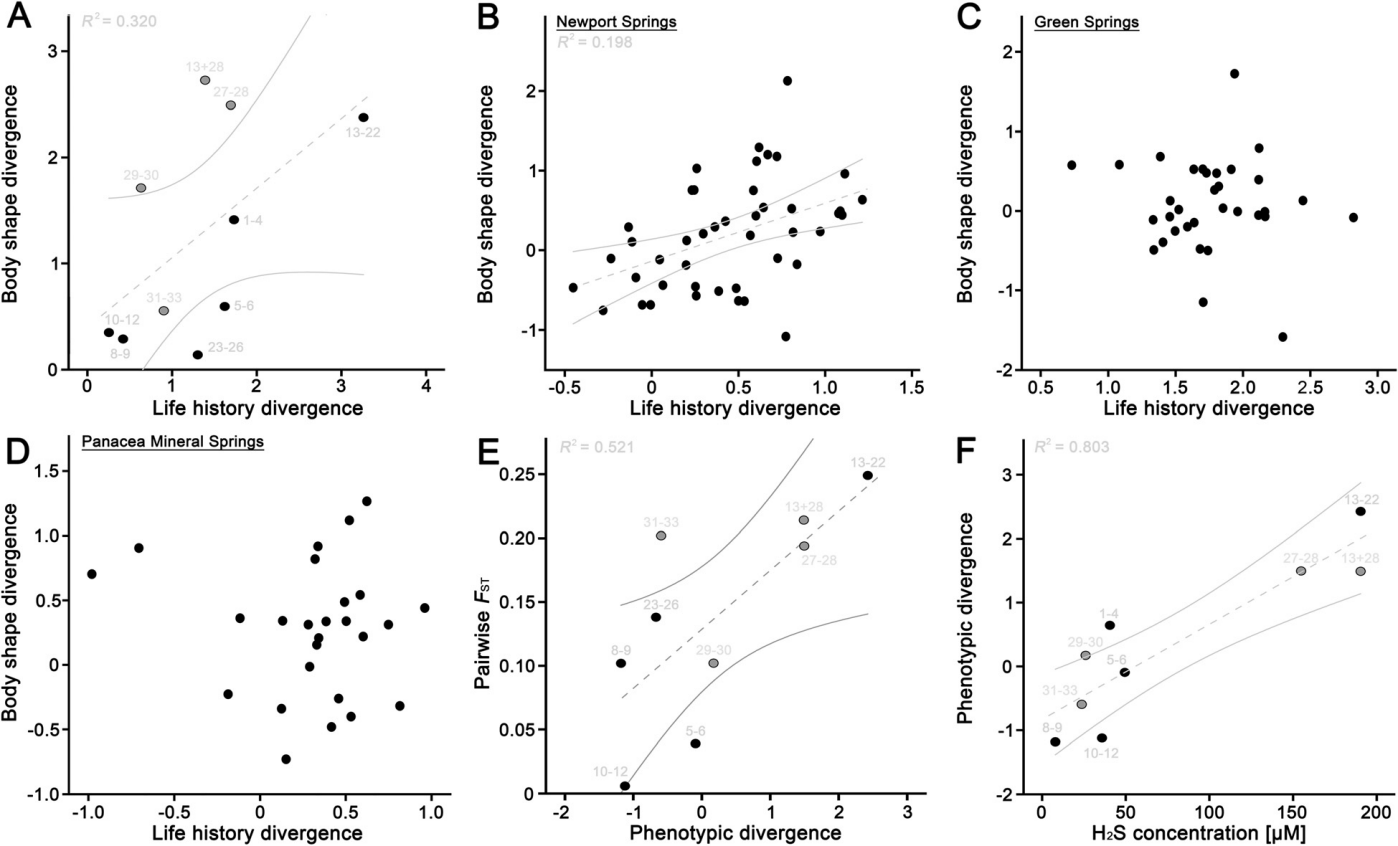
Scatterplots of body shape vs. life-history divergence vector scores (derived from the H_2_S-term in the MANCOVAs) for (**a**) each sulfidic complex, and (**b**–**d**) pregnant female *G. holbrooki* from the three sulfide springs (sites 5, 8 and 10) in Florida. (**e**) Relationship between pairwise *F*_ST_ (as a proxy for gene-flow, see Additional file 1: OSM 6) and phenotypic divergence (data for *G. affinis* not available) and (**f**) relationship between phenotypic divergence and H_2_S-concentrations (data for *G. hubbsi* not available). Black circles represent sulfidic systems inhabited by *Gambusia*, grey circles represent sulfidic systems inhabited by *Poecilia*; numbers correspond to sites as described in Table 1. Regression lines and 95 % CIs in grey are shown for significant correlations (see main text)

Life-history divergence in offspring-related traits was correlated with morphological divergence across all extremophile poeciliid complexes (*N* = 10, *r*_S_ = 0.58, one-tailed *P* = 0.041; Fig. 4a). We further found an individual-level correlation between offspring-related life histories and body shape for *G. holbrooki* females from one of Florida’s three sulfide springs (population 10, Newport Springs: *N* = 44, *r*_P_ = 0.45, one-tailed *P* = 0.001; Fig. 4b), but a similar correlation was lacking from Florida’s other two sulfide-spring populations (population 5, Green Springs: *N* = 33, *r*_P_ = -0.10, one-tailed *P* = 0.29; population 8, Panacea Mineral Springs: *N* = 26, *r*_P_ = -0.11, one-tailed *P* = 0.30, Fig. 4c-d; all three populations combined: *N* = 103, *r*_P_ = -0.003, one-tailed *P* = 0.49). Congruently, a post-hoc ANCOVA on life-history divergence vector scores that included body shape divergence vector scores as a covariate revealed that the slopes for life-history divergence vs. morphological divergence differed significantly between the three populations as indicated by the significant interaction effect (site: *F*_2,97_ = 120.15, *P* < 0.0001; body shape: *F*_1,97_ = 0.31, *P* = 0.58; site × body shape: *F*_2,97_ = 3.64, *P* = 0.03).

We also uncovered a significant correlation between phenotypic divergence and our proxy for gene flow (population genetic differentiation, *F*_ST_: *N* = 9, *r*_S_ = -0.63, one-tailed *P* = 0.035; Fig. 4e), and the degree of phenotypic divergence for each sulfidic site increased with increasing H_2_S-concentration (*N* = 9, *r*_S_ = 0.85, one-tailed *P* = 0.002; Fig. 4f).

## Discussion

Our present study leveraged replicated ecological gradients inhabited by poeciliid fishes of the genera *Gambusia* and *Poecilia*, and examined the speciation continuum using a multivariate approach by simultaneously considering life-history, morphological, and genetic differentiation, and the degree of reproductive isolation in different populations that have colonized H_2_S-rich waters. We uncovered strong signals of both shared and unique aspects of divergence in response to toxic H_2_S across lineages and populations. Specifically, we found (1) some broad patterns of predictable and convergent evolution of offspring-related life histories and adult body shape, (2) potential concerted evolution of different character suites, and (3) that reproductive isolation might emerge as a consequence of local adaptation to sulfidic environments. In other words, this study found evidence consistent with adaptation constraining gene flow, as trait divergence was negatively correlated with gene flow and positively correlated with environmental H_2_S concentrations, and genetic divergence increased between adjacent sulfidic and non-sulfidic populations in 4 of the 5 *Gambusia* systems examined here. To the extent that increased genetic divergence represents reduced gene flow (see Additional file 1: OSM 6 and Figure S6)—a scenario supported by the geographical proximity of the populations compared here—these results suggest that stronger divergent selection drives reduced gene flow in sulfidic systems inhabited by poeciliid fishes (see also [9]). However, we cannot fully exclude the alternative, but not mutually exclusive, direction of causation, whereby gene flow is partially responsible for constraining phenotypic divergence (e.g., [50, 51]).

We did not find any evidence for convergence across clades and toxic environments in adult life histories. Unlike offspring life histories and body shape, adult life-history traits do not seem to experience consistent directional selection from H_2_S, or at least do not exhibit consistent responses to such selection [24, 38]. Instead, the significant effects of sex, clade and site(clade × H_2_S) suggest that variation in adult life histories is likely shaped by other, often population-specific, factors, such as resource availability, sexual conflict and sexual selection or demography, or it reflects genetic drift [52].

### Shared and unique aspects of phenotypic divergence and neutral genetic differentiation in extremophile *Gambusia*

We predicted extremophile mosquitofish to have larger but fewer offspring, and to develop larger heads compared to their closest relatives from non-sulfidic habitats. We uncovered strong overall convergence of morphologies and offspring-related life histories. Across four clades and six sulfur systems, we generally found phenotypic divergence consistent with our a priori predictions of local adaptation to H_2_S-rich environments (question 1).

There were, however, notable exceptions (see Additional file 1: OSM 6 for discussion of some minor patterns of unique divergence). Specifically, extremophile *G. holbrooki* from the Florida Panhandle (populations 8–12) displayed predictable, albeit weak, morphological divergence, but little to no life-history and neutral genetic divergence [i.e., population means for offspring size at birth and fecundity did not differ between populations from sulfidic and non-sulfidic waters in this region (Additional file 1: Table S1)]. The significant effect of H_2_S on offspring-related life histories in our multivariate models, however, indicates that at earlier developmental stages offspring size does indeed differ, but different degrees of embryonic weight loss result in more or less equally sized neonates. This is in contrast to a recent study demonstrating convergence of these traits in sulfidic waters in different lineages of poeciliid fishes [41]. Several non-mutually exclusive mechanisms could account for this observation: First, gene flow in this system might constrain local adaptation, as the spring complex at Panacea Mineral Springs (population 8) is only about 4 × 4 m wide before it drains into a large river where H_2_S immediately gets diluted; this habitat might be too small to allow for a self-sustaining, locally-adapted population (support for this comes from the fact that in the summer of 2015 we only caught *P. latipinna* here but no *G. holbrooki*). Second, H_2_S concentrations at Panacea Mineral Springs were relatively low (see Additional file 1: OSM 1) and might not be sufficient to drive local adaptation and the evolution of reproductive isolation. This hypothesis would also be congruent with the observed positive correlation between phenotypic divergence and H_2_S-concentration, as well as the negative correlation between phenotypic divergence and rates of gene flow. Nonetheless, this hypothesis alone does not explain why a similar pattern was observed at Newport Springs (population 10), which has H_2_S-concentrations apparently high enough to facilitate strong population divergence in *G. affinis* from Oklahoma (populations 1–4) as well as *Poecilia mexicana* from southern Mexico (Additional file 1: OSM 1; Fig. 4f) [9, 24, 34]. Third, while knowledge of the initial stages of ecological speciation in sulfidic habitats remains scarce, one possible scenario outlines a stepwise process: Initial colonizers might move back and forth between sulfidic and non-sulfidic waters, and gradually, subsequent generations of colonizers spend more and more time in sulfidic waters until populations become permanent residents. If this scenario were true, then it is likely that morphological changes evolve first because they are known to have a direct impact on survival of the colonizing individual, while offspring could still be birthed outside of the toxic waters during the early stages of colonization. Given the shallow genetic structure in these particular sulfur systems (see below), one could indeed argue that populations in the Florida Panhandle (populations 8–12) represent the very early stages of diversification in response to H_2_S. Fourth, speciation reversal (i.e., movement towards panmixis) can be initiated when environmental gradients break down or are overridden by another, stronger selective force (e.g., [11–13, 15]). The Florida Panhandle is routinely impacted by tropical storms/hurricanes [53], and accompanying large-scale flooding may lead to temporary population admixture that resets population divergence at regular intervals. However, we consider this explanation to be least likely because the East Coast of Florida and the Bahamas are equally affected by these storms [54], yet population divergence is much stronger. Additionally, we found that patterns of genetic differentiation in sulfidic systems in southern Mexico were not affected by a catastrophic flooding event [36].

### Concerted evolution of life histories and morphologies in *Gambusia* and *Poecilia*

We found that the magnitude of divergence in life history and morphology co-varied with one another across the ten sulfidic complexes (i.e., between-population correlation), and on an individual level both trait suites also co-varied in one out of three *G. holbrooki* sulfide spring populations (i.e., within-population correlation). Furthermore, overall phenotypic divergence also increased with increasing neutral genetic differentiation (a proxy for gene flow) as well as with increasing environmental H_2_S-concentrations (questions 2 and 3).

Positive within-population correlations between life-history and morphological divergence should be indicative of genetic linkage, pleiotropic effects, or developmental interdependencies as driving forces behind the apparent concerted evolution of both character suites; however, we only uncovered this correlation in one out of three populations from sulfidic waters for which data for both character suites were available for the same individuals. While this suggests that genetic linkage, pleiotropy or developmental interdependencies do not play a pivotal role in driving the observed patterns in body shape and offspring-related life histories, future studies using more population replicates will have to investigate this further.

Given the between-population correlation between morphological and life-history divergence, we argue that the presence of H_2_S creates—and is correlated with—complex environmental gradients. These exert multifarious selection that acts strongly and predictably on both genetically- and developmentally-independent character suites (see also [55]). We found additional support for this interpretation, because mean phenotypic divergence across study sites increased as a function of average H_2_S-concentrations.

Why exactly does the strength of phenotypic divergence correlate so strongly with H_2_S-concentration? First, our data indicate that population divergence in sulfidic habitats does not involve major threshold effects (with the possible exception of the comparatively low H_2_S-concentrations present at Panacea Mineral Springs, population 8), but rather mirror a gradual increase in divergent selection with increasing H_2_S-concentration. Our results leave open the possibility that a linear increase in phenotypic divergence occurs with increasing H_2_S concentration until a sufficiently high concentration is reached, after which phenotypes (and presumably selection) change little (i.e., possible asymptotic relationship in Fig. 4e). However, this is difficult to assess in nature, as sites with intermediate H_2_S concentrations are not currently known. Second, the observed phenotypic changes in toxic habitats likely come with associated costs. For example, females experience selection to maximize offspring number per clutch [56], which, all else being equal (i.e., barring other morphological changes that increase body cavity space), is constrained by selection to increase offspring size in sulfidic sites at least to the minimum size needed for efficient H_2_S-detoxification at the given H_2_S-concentration [41–43]. On the morphological side, larger heads are known to reduce fast-start locomotor performance in poeciliid fishes [57, 58], which reduces escape ability and increases vulnerability to fast-striking predators like birds and fishes, and bird predation is known to be strong in many sulfidic habitats [59]. Thus, the morphological changes known to improve respiration in toxic habitats simultaneously render these sulfide-adapted fishes more vulnerable to predators.

### Concerted evolution of phenotypic differentiation and reproductive isolation?

We found that population pairs with greater phenotypic differences exhibited reduced evidence of gene flow (estimated via genetic differentiation), and that phenotypic differences increased with increasing environmental H_2_S-concentrations. Therefore, when focusing on the interplay between phenotypic trait divergence, gene flow, and environmental H_2_S-concentrations, our data suggests that progress along the speciation continuum in extremophile poeciliids is generally promoted by increasing strength of divergent selection associated with higher H_2_S concentrations. In other words, higher H_2_S concentrations lead to stronger and more predictable phenotypic divergence, which in turn results in increased population genetic differentiation (and decreased gene flow), and thus shifts populations further along the speciation continuum towards complete reproductive isolation. This is in line with previous findings in extremophile *Poecilia* suggesting that local adaptations in life-history traits and morphology contribute to reproductive isolation, because they ought to result in a higher relative fitness of locally adapted resident fish compared to immigrants of the alternative ecotype (after [18]; e.g., [9, 33, 60]). This would also support our hypothesis (see above) that selection at Panacea Mineral Springs might simply be too weak to elicit pronounced population divergence. Nonetheless, future experimental data on pre- and post-mating isolation would likely help strengthen these results and yield new insights into the types of reproductive isolation promoting genetic divergence in each system.

For species and populations inhabiting environments characterized by H_2_S-concentrations ranging between ca. 30 and 40 μM (i.e., *G. affinis* from Vendome Well and *G. holbrooki* from Green Springs and Newport Springs in Florida; populations 1, 5, and 10, respectively), we uncovered substantial variation in the degree of phenotypic divergence and neutral genetic differentiation, indicating population-specific variation in the progress towards complete reproductive isolation. Several non-mutually exclusive scenarios are plausible that might help explain these differences. For example, if all of these populations do in fact experience the same speciation mechanisms, then populations undergoing divergent selection or genetic drift for longer should have moved further along the speciation continuum than populations that diverged more recently [7, 8, 16]. Furthermore, while it is tempting to assume that divergence in H_2_S-rich waters takes place in parapatry (based on the present situation found in sampled habitats), we cannot exclude the possibility that most systems that exhibit advanced stages of phenotypic divergence and neutral genetic differentiation have undergone at least temporary phases of allopatry during which populations from surrounding non-sulfidic waters temporarily collapsed. Evidence for this comes from southern Mexican sulphur mollies (*Poecilia sulphuraria*), which are genetically more closely related to northern Mexican *P. mexicana limantouri* than the southern Mexican *P. mexicana mexicana* they currently share the same continuous habitat with [34, 40, 61]. Moreover, H_2_S-discharge might be more temporally variable in some populations than others, with advanced divergence only being present in habitats that experience more constantly elevated H_2_S-concentrations. Finally, additional selective agents may be at play in some populations that either counteract or enhance H_2_S-induced selection, or this could result from mutation-order speciation, differences in the genetic architecture of the founder populations, and/or stochastic effects [7, 8, 16].

## Conclusions

Our study highlights the diversity and complexity of organismal responses to immediate and evolutionary exposures to stressors, despite the fact that some physicochemical stressors (like H_2_S) appear to have clear-cut and predictable physical, chemical, or physiological effects on biological and non-biological processes at all levels of organization. This suggests an environmental complexity associated with the presence of physicochemical stressors, which can cause multifarious selective regimes through direct and indirect effects on abiotic and biotic components of an ecosystem. Such environmental complexity interacting with phenotypic integration and variation in genomic architecture can result in a pattern of shared and unique organismal responses similar to the one revealed here for extremophile poeciliids (with emphasis on *Gambusia* spp.) inhabiting sulfidic waters. Hence, integrative analyses of environmental and phenotypic complexity promise to gain a new level of sophistication in our understanding of life in extreme environments.

## Methods

### Study sites and sample collections

We collected mosquitofishes and mollies in sulfidic and adjacent non-sulfidic habitats in Oklahoma (*G. affinis*), Florida (*G. holbrooki*), Mexico (*G. sexradiata*, *G. eurystoma, P. mexicana,* and *P. sulphuraria*), and The Bahamas (*G. hubbsi*; Fig. 1; Table 1; see Additional file 1: Online Supplementary Material (OSM) 1 and 2 for details). All specimens designated for life-history and morphological analyses were preserved in 10 % formaldehyde solution, while specimens/fin clips collected for molecular genetic analyses were preserved in 70–95 % ethanol. For four species, we collected populations from both sulfidic and non-sulfidic habitats; however, this was not possible for *G. eurystoma* and *P. sulphuraria*, which are endemic to sulfide spring complexes in Tabasco, southern Mexico [62]. Both were therefore compared to their closely related sister species from non-sulfidic waters surrounding the habitats of the sulfide-spring endemics: *G. eurystoma* was compared to *G. sexradiata* [41, 63], and *P. sulphuraria* was compared to *P. mexicana* [40, 41]. Within each system, the degree of physical (geographical) separation between divergent habitats (i.e., sulfidic and non-sulfidic) is similar to, or frequently less than, that between similar (i.e., non-sulfidic) habitats (Fig. 4). Therefore, geographic isolation is not confounded with habitat type. Morphological data for *G. affinis*, *P. mexicana*, and *P. sulphuraria* were extracted from photographs used for previously published studies [40, 47]; however, we changed the original landmark configuration to match the one applied to all *Gambusia* spp. in the present study (Additional file 1: Figure S1). Offspring size at birth and fecundity data for most *Gambusia* and *Poecilia* populations were re-analyzed from [24, 41] (see Table 1 for details). All other data were collected specifically for this study.

### Life-history and morphometric analyses

Dissections to collect male, female, and offspring-related life-history traits followed well-established protocols (e.g., [37, 38, 64]. We collected the following male and female life-history traits: standard length (SL [mm]), dry weight (g), lean weight (g), fat content (%), and reproductive investment [%; for males: testis dry weight divided by the sum of reproductive tissue dry weight and somatic dry weight (gonadosomatic index, GSI); for females: offspring dry weight divided by the sum of offspring dry weight plus somatic dry weight (reproductive allocation, RA)]. We note that GSI for males and RA for females do not capture total investment into reproduction, as it obviously ignores other costs of reproduction, such as energetic costs related to searching for mates, sneak-mating, and intrasexual competition for mates [65]. This is particularly true for males, where the relative behavioral costs of reproduction may exceed those captured by GSI [66]. Thus, patterns in total reproductive costs may not necessarily mirror those estimated via GSI and RA.

For females we also collected data on fecundity (number of developing offspring), offspring dry weight (mg), offspring lean weight (mg), and offspring fat content (%). Prior to statistical analyses we log_10_-transformed (male and female SL, male and female lean weight, and embryo dry and lean weight), square root-transformed (fecundity), or arcsine(square root)-transformed (male and female fat content, male and female GSI, embryo fat content) all life-history variables, and conducted subsequent *z*-transformation to meet assumptions of statistical analyses (i.e., these transformations facilitated normality of model residuals).

To quantify morphological differentiation, lateral photographs were taken using Canon DSLR cameras with a macro lens, and we then digitized 13 landmarks on each image (Additional file 1: Figure S1) using the software program tpsDig2 [67]. We performed geometric morphometric analysis as described in [32, 34] (see Additional file 1: OSM 3 for details), resulting in seven principal component axes (= relative warps) explaining 91.5 % of the morphological variance as variables for the statistical analyses.

### Phenotypic differentiation based on life histories and body shape

We first tested for differences in adult body size (SL) between populations by conducting mixed-model analysis of variance (ANOVA) that included the following independent variables: clade (four levels: *G. affinis*, *G. holbrooki*, *G. eurystoma/G. sexradiata*, and *G. hubbsi*), sex, H_2_S (present or absent), and “site nested within clade-by-H_2_S” [random effect, hereafter: site(clade × H_2_S)]. In this and all subsequent statistical models we initially included all potential two-way and three-way interactions, but removed terms from the final model in a step-wise process if *P* > 0.1, with the exception that a term with *P* > 0.1 would be retained if a higher-order interaction term involving that term had a *P* < 0.1. We then conducted three separate mixed-model multivariate analyses of covariance (MANCOVA) examining variation in adult life history, offspring life history, and body morphology (see below for model structure). Assumptions of multivariate normal error distribution and homogeneity of variances and covariances were met for all analyses performed. Statistical significance was determined using an *F*-approximation from Wilks’s lambda for all model terms with the exception that we used an *F*-test using restricted maximum likelihood and the Kenward-Roger degrees of freedom adjustment [68] for clade, H_2_S, and clade × H_2_S to appropriately test these fixed effects while treating site as a random term (i.e., effectively treating site as the unit of replication; see also [69, 70]). The latter significance test was conducted using the MIXED procedure in SAS (SAS Institute, Cary, NC; sample code in the appendix of [69]), while all other tests were conducted in JMP (SAS). To quantify the relative importance of model terms, we calculated effect size using Wilks’s partial eta squared (*η*_p_^2^) and calculated the relative variance as the partial variance for a given term divided by the maximum partial variance value in that model.

The first model tested for differentiation based on adult life histories and included lean weight, fat content, and GSI as dependent variables. To control for multivariate allometry, standard length (SL) was added as a covariate, and we included clade, sex, and H_2_S as fixed factors, and site(clade × H_2_S) as a random effect. The second model tested for differentiation based on offspring-related life histories and included fecundity, embryo lean weight, and embryo fat content as dependent variables. The fixed factors for this model were clade and H_2_S, while site(clade × H_2_S) was included as a random effect, and the covariates were SL and ‘embryonic stage of development’ (see [39] for details on embryo stages). The third model tested for phenotypic differentiation among sites based on body shape variation, using the seven retained relative warps as dependent variables. We tested for effects of centroid size to control for multivariate allometry and included clade, sex, and H_2_S as fixed factors, and site(clade × H_2_S) as a random effect. Running the analyses of adult life histories and body shape variation for both sexes separately confirmed our results and interpretations presented here (results not shown).

To assess the nature and strength of convergent life-history and morphological divergence in response to H_2_S among clades and sites, we performed a canonical analysis of the H_2_S-term of each MANCOVA to derive divergence vectors following [71]. Each divergence vector describes the linear combination of dependent variables that exhibits the greatest difference between habitat types in Euclidean space, while controlling for other terms in the model (see Additional file 1: OSM 4 for details). We visualized shape variation described by the H_2_S term included in the MANCOVA with thin-plate spline transformation grids using tpsRegr [72].

### Population genetic analyses

We used 11 nuclear microsatellite loci to genotype *N* = 382 fish from 17 sites (Table 1; Fig. 4) in all species except *G. affinis* (for which no alcohol-preserved material was available, see Additional file 1: OSM 1) using previously established protocols [73, 74] (see Additional file 1: OSM 5 for details). We used ARLEQUIN v 3.5 [75] to calculate pairwise *F*_ST_-values between populations in each drainage and to calculate standard indicators of genetic variability (see Additional file 1: OSM 5). STRUCTURE v 2.3.4 [76] was employed to identify the number of genetically distinct clusters (*K*) in each drainage with the method outlined by [77] using the web-based tool STRUCTURE HARVESTER v 0.6.8 [78]. Ten iterations per *K* were run using the admixture model with a burn-in period of 10^6^ generations, followed by 10^6^ iterations for *K* = 1 up to twice the number of sampling sites included per area. Each simulation was performed using an ancestry model incorporating admixture, a model of correlated allele frequencies, and no prior information on locations.

To test whether divergent selection between sulfidic and non-sulfidic sites was associated with population genetic structure (i.e., higher *F*_ST_) while controlling for phylogenetic differences between clades, we employed a partial Mantel test with pairwise *F*_ST_-values obtained from FSTAT v 2.9.3 [79] as dependent variable, habitat difference as independent variable (0 = same habitat type, 1 = different habitat type), and clade as the covariate (0 = same clade, 1 = different clade). For this analysis, we compiled a global dataset including all *Gambusia* species for which microsatellite data were available (i.e., without *G. affinis*). To standardize comparisons across clades, we only included up to a maximum of two non-sulfidic sites for each sulfidic system in the first model, so that the final dataset consisted of the populations 5–12 for *G. holbrooki*, 13 and 16–17 for *G. eurystoma*/*G. sexradiata*, and 23–24 and 26 for *G. hubbsi* (see Table 1 for more details). Due to the lack of neutral genetic differentiation in the populations from the Florida Panhandle (see results), we conducted a second partial Mantel test that excluded those populations.

### Joint evolution of life histories, morphologies, and reproductive isolation?

To increase sample size and statistical power, most of the following analyses were conducted on fishes from both genera (*Gambusia* and *Poecilia*; Fig. 4), thus adding offspring-related life-history data (*N* = 215), body shape data (*N* = 539), and population genetic data (*N* = 174) for *Poecilia* spp. from four additional sulfide spring systems (Table 1; Additional file 1: OSM 2 and Table S6 for details).

To investigate patterns of correlated phenotypic divergence across sulfide spring systems, we calculated average offspring-related life-history and morphological divergence scores for each sulfide spring complex (i.e., average divergence vector score for the non-sulfidic habitats – divergence vector score for the sulfide spring) and compared them with a Spearman rank correlation. We only included these two character suites as our results indicated that they exhibited the strongest shared responses to H_2_S (see results section). For *Poecilia* spp., we calculated scores along the divergence vectors derived for *Gambusia* spp. (described above) by projecting those individuals onto the relevant multivariate axes using the eigenvector coefficients.

We further investigated if the correlation between morphological and offspring-related life-history divergence can also be found on the individual level. For this we used a subset of the data, for which we had collected both life-history and morphological data from the same individuals [i.e., this included only pregnant *G. holbrooki* females from the three sulfide springs in Florida (sites 5, 8 and 10)]. The predicted positive correlation between variables (here and in subsequent correlations) was tested using one-tailed tests; non-parametric tests we used where appropriate to account for non-normally distributed data.

To create a single variable of phenotypic divergence, we subjected average life-history and morphological divergence scores for each sulfide system to a PCA based on a correlation matrix, and one principal component with an eigenvalue of 1.88 was retained, accounting for 94.0 % of the total variance. To investigate the relationship between overall phenotypic divergence and reproductive isolation, we calculated a Spearman rank correlation between these PC1 scores and pairwise *F*_ST_-values between a given sulfide spring and the geographically closest non-sulfidic site (*F*_ST_-values represented a good proxy for gene-flow in our system; see Additional file 1: OSM 6 for details). Due to a lack of population genetic data, *G. affinis* was excluded from this analysis.

Finally, we asked if the degree of ecologically-based divergent selection (approximated by average, system-specific H_2_S-concentrations) predicts the degree of phenotypic divergence. Since no data on H_2_S-concentrations for the *G. hubbsi* complex from the Bahamas were available, that clade was excluded from the analysis. We then tested for a correlation between PC scores for total phenotypic divergence (see above) and average H_2_S-concentrations of that particular sulfidic site using another Spearman rank correlation.

## Additional file

Additional file 1: OSM 1: Additional information on *Gambusia* collection sites. (DOCX 2179 kb)
