# Supplementary material for: Extremophile Poeciliidae: multivariate insights into the complexity of speciation along replicated ecological gradients
Source: BMC Evol Biol. 2016 Jun 22;16:136. doi: 10.1186/s12862-016-0705-1 (PMC4918007; doi:10.1186/s12862-016-0705-1)

**Supplement to**

**Extremophile Poeciliidae: multivariate insights into the complexity of speciation**

**along replicated ecological gradients**

**R. Riesch, M. Tobler, H. Lerp, J. Jourdan,**

**T. Doumas, P. Nosil, R.B. Langerhans, M. Plath**

**OSM 1: Additional Information on *Gambusia* Collection Sites**

We collected western mosquitofish (*Gambusia affinis*) from the sulfide spring complex at Vendome Well (H_2_S-concentration, mean ± s.d.: 40.5 ± 30.6 µM) in the Chickasaw National Recreation Area near the town of Sulphur in Oklahoma (Covich 1981; Tobler and Hastings 2011). Reference samples from non-sulfidic habitats were obtained from the adjacent Travertine Creek just upstream of the confluence with Vendome Well, as well as from several creeks throughout Oklahoma (see main manuscript, Fig. 1A and B; Table 1). Life-history collections were made in July 2009, and samples for morphology were collected in September 2007. Due to permit restrictions within Chickasaw National Recreation Area, no samples for population genetic analyses could be obtained. Morphological data were re-analyzed from a previous study (Tobler and Hastings 2011).

Eastern mosquitofish (*Gambusia holbrooki*) were collected from three independent sulfide spring complexes in Florida (see main manuscript, Fig. 1A and C; Table 1). Green Springs (49.4 ± 3.2 µM H_2_S) is located in Green Springs Park in Volusia County, and reference collections were made in the adjacent Lake Monroe (into which the Green Springs brook drains) as well as in a flood drain in the city of Melbourne. Panacea Mineral Springs (7.9 ± 2.6 µM H_2_S) is an old spa complex at Dickerson Bay in Wakulla County, and we obtained a reference collection from a drainage ditch to the southwest of Carabelle. Newport Springs (35.8 ± 4.8 µM H_2_S) is located just north of Newport in Wakulla County, and reference collections were made in the St. Marks River just south of the confluence with the waters originating from Newport Springs as well as in a drainage ditch in St. Marks (main manuscript, Fig. 1A and C; Table 1). Specimens designated for life-history and morphological analyses were collected in May 2012, while collections for population genetic analyses were made in August 2011.

Teardrop mosquitofish (*Gambusia sexradiata*) are widespread along the Atlantic slope of southern Mexico (Miller 2005). Nested within the distribution of *G. sexradiata* is the geographic distribution of its sister taxon, the widemouth mosquitofish (*Gambusia eurystoma*; Lydeard et al. 1995). This species is endemic to a small, highly sulfidic stream at the Baños del Azufre (190.4 ± 119.7 µM H_2_S), which is part of the Pichucalco river drainage in the states of Tabasco and Chiapas (Miller 2005; Tobler et al. 2008). We obtained reference collections of *G. sexradiata* from various sites along the Pichucalco river drainage. Due to permit restrictions, life-history collections were made in January 2009 (*G. sexradiata* and female *G. eurystoma*) and January 2010 (male *G. eurystoma*), while collections for morphological and population-genetic analyses were obtained in July and August 2011.

We collected Bahamas mosquitofish (*Gambusia hubbsi*) on northern Andros Island. Specimens from a sulfidic habitat were collected from Archie’s Blue Hole (H_2_S-concentration not available). Blue holes are characterized by a freshwater lens that floats on top of denser salt water; the interface between both water bodies is called the halocline, and it is here that bacteria produce H_2_S (Todhunter 2010). While the freshwater lens can reach a depth of many meters in some blue holes, it is only a few dozen centimeters thick in Archie’s Blue Hole (Langerhans, unpublished data). Reference samples were collected from three nearby tidal creeks (see main manuscript, Figs. 1A and E; Table 1). We collected specimens designated for life-history and morphological analyses in August 2002, while collections for population genetic analyses were made in March 2010.

*Literature Cited*

Covich, A. 1981. Chemical refugia from predation for thin-shelled gastropods in a sulfide-enriched stream. Verhandlungen der Internationalen Vereinigung fuer Limnologie 21:1632–1636.

Lydeard, C., M. C. Wooten and A. Meyer. 1995. Cytochrome b sequence variation and a molecular phylogeny of the live-bearing fish genus *Gambusia* (Cyprinodontiformes: Poeciliidae). Can. J. Zool. 73:213–227.

Miller, R. R. 2005. Freshwater fishes of Mexico. University of Chicago Press, Chicago.

Tobler, M. and L. Hastings. 2011. Convergent patterns of body shape differentiation in four different clades of poeciliid fishes inhabiting sulfide springs. Evol. Biol. 38:412–421.

Tobler, M., R. Riesch, F. J. García de León, I. Schlupp and M. Plath. 2008b. Two endemic and endangered fishes, *Poecilia sulphuraria* (Alvarez, 1948) and *Gambusia eurystoma* Miller, 1975 (Poeciliidae, Teleostei) as only survivors in a small sulphidic habitat. J. Fish Biol. 72:523–533.

Todhunter, A. 2010. Deep dark secrets. National Geographic 8:34–53.

**OSM 2: Additional Information on *Poecilia* Collection Sites**

We collected *Poecilia* spp. with a seine (4 m long, 4 mm mesh-width) in the vicinity of the city of Teapa in Tabasco, southern Mexico (see Palacios et al. 2013 for a map of the sampling region). Sulfide spring complexes inhabited by *Poecilia* spp. are located in the foothills of the Sierra Madre de Chiapas and are distributed across three major tributaries of the Río Grijalva (from east to west: Ríos Tacotalpa, Puyacatengo, and Pichucalco). Non-sulfidic and sulfidic habitats within each of the drainages are interconnected and not separated by physical barriers that would prevent fish migration. All three rivers eventually join the Río Grijalva and are widely interconnected in the lowlands during the wet season; however, the sulfidic spring complexes are separated by mountains (Miller 1966). H_2_S in springs of this region stems from bacterial sulfate reduction in aquifers fed by meteoric water (Rosales Lagarde 2012). Sulfur and carbon sources to fuel bacterial metabolism are associated with hydrocarbon deposits (Rosales Lagarde 2012) and potentially volcanic activity (Rosales Lagarde et al. 2006).

Within the Río Tacotalpa drainage, Atlantic mollies (*Poecilia mexicana*) were collected from the sulfidic El Azufre I (H_2_S-concentration, mean ± s.d.: 23.7 ± 18.2 µM), and reference samples were obtained from the non-sulfidic Arroyo Bonita and Río Amatan. All of these collection sites are located in the vicinity of the small town of Tapijulapa, in southern Tabasco. Life-history and morphological data for these populations were re-analyzed from previous studies (Riesch et al. 2010, Palacios et al. 2013). Population genetic data were re-analyzed from Plath et al. (2013).

Within the Río Puyacatengo drainage, we collected *P. mexicana* from the sulfidic spring at La Lluvia (26.2 ± 18.3 µM H_2_S), which drains into the Río Puyacatengo, from which we collected our reference samples from non-sulfidic waters. Life-history samples were collected in the summer of 2010 and comprised *N* = 15 and *N* = 31 pregnant females from La Lluvia and Río Puyacatengo, respectively. Morphological and population genetic data were re-analyzed from previous studies (Palacios et al. 2013; Plath et al. 2013).

Atlantic mollies are also widespread in the Río Pichucalco drainage. Nested within the distribution of *P. mexicana* is the geographic distribution of its sister taxon, the sulphur molly (*Poecilia sulphuraria*; Palacios et al. 2013; Pfenninger et al. 2014). This species is endemic to at least two small, highly sulfidic spring complexes at the Baños del Azufre (190.4 ± 119.7 µM H_2_S) and Rancho La Gloria (154.8 ± 54.5 µM H_2_S), in the states of Tabasco and Chiapas (Tobler et al. 2011). We obtained reference collections of *P. mexicana* from a single site within the Pichucalco river drainage, Arroyo Rosita. Life-history collections for La Gloria and Arroyo Rosita were made in the summer of 2010 (*N* = 27 and *N* = 37 pregnant females, respectively), while *P. sulphuraria* life histories were re-analyzed from a previous study (Riesch et al. 2010). We re-analyzed morphological data from Palacios et al. (2013), and population genetic data from Slattery et al. (2012).

*Literature Cited*

Miller, R. R. 1966. Geographical distribution of Central American freshwater fishes. Copeia 1966:773–802.

Palacios, M., L. Arias-Rodriguez, M. Plath, C. Eifert, H. Lerp, A. Lamboj, G. Voelker, and M. Tobler. 2013. The rediscovery of a long described species reveals additional complexity in speciation patterns of poeciliid fishes in sulfide springs. PLoS ONE 8:e71069.

Pfenninger, M., H. Lerp, M. Tobler, C. Passow, J. L. Kelley, E. Funke, B. Greshake, U. K. Erkoc, T. Berberich, and M. Plath. 2014. Parallel evolution of cox genes in H_2_S-tolerant fish as key adaptation to a toxic environment. Nat. Commun. 5:3873.

Plath, M., M. Pfenninger, H. Lerp, R. Riesch, C. Eschenbrenner, P. A. Slattery, D. Bierbach, N. Herrmann, M. Schulte, L. Arias–Rodriguez, J. R. Indy, C. Passow, and M. Tobler. 2013. Genetic differentiation and selection against migrants in evolutionarily replicated extreme environments. Evolution 67:2647–2661.

Riesch, R., M. Plath, F. García de León, and I. Schlupp. 2010. Convergent life-history shifts: toxic environments result in big babies in two clades of poeciliids. Naturwissenschaften 97:133–141.

Rosales Lagarde, L., P. J. Boston, A. Campbell, and K. W. Stafford. 2006. Possible structural connection between Chichón Volcano and the sulfur-rich springs of Villa Luz Cave (a. k. a. Cueva de las Sardinas), Southern Mexico. Assoc. Mex. Cave Stud. Bull. 19:177–184.

Rosales Lagarde, L. P. 2012. Investigation of karst brackish-sulfidic springs and their role in the hydrogeology, subsurface water-rock interactions, and speleogenesis at northern Sierra de Chiapas, Mexico. Ph. D. Dissertation. New Mexico Institute of Mining and Technology: Socorro, NM, USA.

Slattery, P., C. Eschenbrenner, L. Arias-Rodriguez, B. Streit, D. Bierbach, R. Riesch, M. Tobler, M. Pfenninger, B. Feldmeyer, M. Plath, and H. Lerp. 2012. Twelve new microsatellite loci for the sulphur molly (*Poecilia sulphuraria*) and the related Atlantic molly (*P. mexicana*). Cons. Genet. Resour. 4:935–937.

Tobler, M., M. Palacios, L. J. Chapman, I. Mitrofanov, D. Bierbach, M. Plath, L. Arias-Rodriguez, F. J. García de León, and M. Mateos. 2011. Evolution in extreme environments: replicated phenotypic differentiation in livebearing fish inhabiting sulfidic springs. Evolution 65:2213–2228.

**OSM 3: Additional Information on Geometric Morphometric Assessments**

For all specimens, lateral photographs were taken using Canon SLR cameras with a macro-lens, and we then digitized 13 landmarks on each image (Fig. S1) using the software program tpsDig2 (Rohlf 2010a). Landmark coordinates were aligned using least-squares superimposition as implemented in the program tpsRelw (Rohlf 2010b) to remove effects of translation, rotation, and scale. After realignment, we calculated centroid size and partial warp scores with uniform components (weight matrix) for each individual. The weight matrix was subjected to a principal component analysis based on a covariance matrix to reduce data dimensionality (i.e., relative warps analysis). We were limited in the number of dependent variables we could use in our mixed model (because sampling sites served as units of replication); hence, we aimed to retain 90% or more of the shape variance. This resulted in retention of seven principal component axes (relative warps) explaining 91.5% of variance as variables for statistical analyses.

*Literature Cited*

Rohlf, F. 2010a. tpsDig. Available from http://life.bio.sunysb.edu/morph/ (accessed June 30, 2010).

Rohlf, F. 2010b. tpsRelw. Available from http://life.bio.sunysb.edu/morph/ (accessed June 30, 2010).

**OSM 4: Calculating Divergence vectors**

Eigenvectors of divergence (**d**) were calculated following Langerhans (2009), and we used these axes for interpretation of multivariate effects instead of standard canonical variates because the latter introduce distortion of trait space owing to multiplication by a matrix inverse (Langerhans 2009; Mitteroeker and Bookstein 2011; please see below for more details). This procedure represents a canonical decomposition of the H_2_S-term from a particular MAN(C)OVA model, resulting in vectors that aid in interpretation of effects present in a given model. **d** was originally derived to help with the interpretation of morphological landmark data, but to make values comparable across phenotypic traits, we calculated it for life-history data as well [rather than deriving a simple canonical variate from the H_2_S-term (i.e., principal components analysis of **H×E^-1^**, where **H** is the sums of squares and cross-products matrix of the term of interest, and **E^-1^** is the inverse of the error sums of squares and cross-products matrix)]. In general, scaling the multidimensional space by a matrix inverse prior to matrix diagonalization (as with standard canonical analysis) generates some degree of distortion in the multivariate space relative to the original variable space unless within-group variation is isotropic (i.e., **E** ∝ identity matrix), which is typically not the case (see Klingenberg and Monteiro 2005; Mitteroeker and Bookstein 2011). Thus, we performed diagonalization of **H** directly by performing a PCA of **H** (for the H_2_S-term) to derive an eigenvector of divergence (**d**) in phenotype between habitat types (i.e., sulfidic versus non-sulfidic). This technique avoids the distortion caused by the minimization of within-group variation and maximization of between-group variation. Thus, **d** describes the linear combination of dependent variables exhibiting the greatest difference between sulfidic and non-sulfidic environments in Euclidean space, while statistically controlling for other terms in the model. This can also be conceptualized as a between-groups PCA conducted using model-adjusted group means (least-squares means from the MAN(C)OVA, which controls for other model terms) rather than raw group means.

*Literature Cited*

Klingenberg, C. P., and L. R. Monteiro. 2005. Distances and directions in multidimensional shape spaces: implications for morphometric applications. Syst. Biol. 54: 678–688.

Langerhans, R. B. 2009. Trade-off between steady and unsteady swimming underlies predator-driven divergence in *Gambusia affinis*. J. Evol. Biol. 22:1057–1075.

Mitteroecker, P., and F. Bookstein. 2011. Linear discrimination, ordination, and the visualization of selection gradients in modern morphometrics. Evol. Biol. 38:100–114.

**OSM 5: Additional Information on Population Genetic Analyses**

DNA was extracted from ethanol-preserved tissue samples using the NucleoSpin Tissue Kit (Macherey-Nagel, Düren, Germany) according to the manufacturer’s recommendations. We used primer pairs established for *G. affinis* (Spencer et al. 1999; Purcell et al. 2011), which were arranged in three separate multiplex reactions (reaction 1: Gaaf10, Gaaf13, Gafu3; reaction 2: Gaaf7, Gaaf9, Gaaf15, Gaaf16, Gafu2, Gafu6; reaction 3: Gafu1, Gafu7) and amplified using the Type-it Mirosatellite PCR kit (Qiagen, Hilden, Germany) with PCR conditions as follows: initial denaturation for 5:00 min at 95°C, 30 cycles of 1:30 min at 60°C, and 0:30 min at 72°C, final extension for 30:00 min at 60°C. Each 5 µl reaction mix included 2.5 µl Type-it master mix, 0.4 µl primer mix, 0.4 µl Q-solution, 0.9 µl RNase-free water, and 0.8 µl template DNA. Fragment sizes were scored manually after electrophoresis on a Beckman Coulter capillary sequencer CEQ 2000, using an internal size standard (Beckman Coulter).

ARLEQUIN v 3.5 (Excoffier and Lischer 2010) was used to calculate expected (*H*_E_) and observed heterozygosity (*H*_O_), to test for deviations from Hardy-Weinberg-Equilibrium and to calculate pairwise *F*_ST_-values between populations in each drainage. FSTAT v 2.9.3 (Goudet 2001) was used to calculate allelic richness (*A*). We tested for null alleles at each locus using Micro-checker v 2.2.3 (van Oosterhout et al. 2004) for each species separately.

In case of Floridian samples, we conducted a preliminary STRUCTURE analysis using all samples to infer whether a single sulfide-adapted ecotype exists that is distributed in several H_2_S sources across the entire peninsula. Our Bayesian clustering analysis uncovered *K* = 2 as the uppermost hierarchical level of population structure, but the analysis separated southern from northern samples rather than all populations inhabiting sulfidic waters from those inhabiting non-sulfidic waters (not shown in detail). We also tested for isolation by distance using a partial Mantel test with pairwise *F*_ST_-values as dependent variable and geographic distance and habitat difference (0 = same habitat type, 1 = different habitat type) as independent variables using FSTAT v 2.9.3. However, only geographic distance had a significant effect on genetic differentiation (habitat difference: *r* = -0.03, *P* = 0.87; geographic distance: *r* = 0.52, *P* = 0.005)*.* The lack of a significant effect of habitat type in this analysis is simply an effect of the almost non-existent neutral genetic differentiation detected in the two sulfidic sites from the Florida Panhandle (see results in main text), rather than an indication that habitat type in general is not important for population divergence.

Finally, strong selection in sulfidic habitats might result in lowered neutral genetic variance as a result of both population bottlenecks and/or selective sweeps (i.e., elimination of standing variation in regions linked to recently fixed beneficial mutations; Fay and Wu 2000; but see Walsh and Blows 2009), so we further asked if the presence of H_2_S also predicts neutral genetic variance. We therefore tested for reduced neutral genetic variability in populations from sulfidic habitats compared to populations from adjacent non-sulfidic waters by calculating and thus, calculated mean values of allelic richness (*A*) and observed heterozygosity (*H*_O_) for each sulfidic site, and mean values for the two metrics across the nearby non-sulfidic sites in each study area. We compared the values between sulfidic and non-sulfidic sites via Wilcoxon signed rank tests using SPSS 12, SPSS Inc. (Chicago, IL). However, we detected no statistically significant differences in mean allelic richness (Wilcoxon test, *N* = 5, *z* = -0.67, *P* = 0.50) or observed heterozygosity (*H*_o_; *N* = 5, *z* = -0.94, *P* = 0.35; Fig. S3) between toxicity regimes.

*Literature Cited*

Excoffier, L. and H. Lischer. 2010. Arlequin suite ver 3.5: A new series of programs to perform population genetics analyses under Linux and Windows. Mol. Ecol. Resour. 10:564–567.

Fay, J. C., and C.-I. Wu. 2000. Hitchhiking under positive Darwinian selection. Genetics 155:1405–1413.

Goudet, J. 2001. FSTAT, a program to estimate and test gene diversities and fixation indices (version 2.9.3; http://www2.unil.ch/popgen/softwares/fstat.htm).

Purcell, K. M., S. L. Lance, K. L. Jones and C. A. Stockwell. 2011. Ten novel microsatellite markers for the western mosquitofish *Gambusia affinis*. Cons. Genet. Resour. 3:361–363.

Spencer, C. C., C. A. Chlan, J. E. Neigel, K. T. Scribner, M. C. Wooten and P. L. Leberg. 1999. Polymorphic microsatellite markers in the western mosquitofish, *Gambusia affinis*. Mol. Ecol. 8:157–168.

Van Oosterhout, C., W. F. Hutchinsons, D. P. M. Wills and P. Shipley. 2004. Micro-Checker: software for identifying and correcting genotyping errors in microsatellite data. Mol. Ecol. Notes 4:535–538.

Walsh, B., and M. W. Blows. 2009. Abundant genetic variation + strong selection = multivariate genetic constraints: a geometric view of adaptation. Annu. Rev. Ecol. Evol. Syst. 40:41–59.

**OSM 6: Estimates of Gene Flow and their relationship to *F*_ST_**

To investigate if the amount of genetic differentiation (measured via *F*_ST_) can be used as a reliable proxy for gene flow, we calculated two different and independent measures for gene flow using our nuclear microsatellite data. First, we calculated the mean genetic assignment to the nonresident genetic cluster (obtained from a population genetic assignment test in STRUCTURE and averaged for the ten runs for *K* = 2; following Plath et al. 2013) between a given sulfide spring and the geographically closest non-sulfidic site. Second, we calculated first-generation migrants via GENECLASS2 (Piry et al. 2004), using the L_home likelihood computation, the Bayesian method of classification (Rannala and Mountain 1997), and a threshold *P*-value of 0.05 (Figures S4 and S5). For subsequent correlations (see below), we then calculated a mean number of first-generation migrants for each sulfidic system by averaging the number of first-generation immigrants and emigrants between that sulfidic system and the geographically closest non-sulfidic habitat.

Spearman rank correlations between these measures of gene flow and pairwise *F*_ST_-values were highly significant (assignment to non-resident genetic cluster: *N* = 9*,* *r*_S_ = -0.75, one-tailed *P* = 0.010; Fig. S6A; first-generation migrants: *N* = 9*,* *r*_S_ = -0.91, one-tailed *P* < 0.001; Fig. S6B), supporting the interpretation that for extremophile poeciliids diverging in toxic sulfide springs, *F*_ST_-values are indeed a good proxy for gene flow.

*Literature Cited*

Piry, S., A. Alapetite, J. Cornuet, D. Peatkau, L. Baudouin, and A. Estoup. 2004. GENECLASS2: a software for genetic assignment and first-generation migrant detection. J. Hered. 95:536-539.

Plath, M., M. Pfenninger, H. Lerp, R. Riesch, C. Eschenbrenner, P. A. Slattery, D. Bierbach, N. Herrmann, M. Schulte, L. Arias–Rodriguez, J. R. Indy, C. Passow, and M. Tobler. 2013. Genetic differentiation and selection against migrants in evolutionarily replicated extreme environments. Evolution 67:2647–2661.

Rannala, B., and J. Mountain. 1997. Detecting immigration by using multilocus genotypes. Proc. Natl Acad. Sci. USA 94:9197-9221.

**OSM 7: Additional Discussion of some population-specific patterns of unique divergence in response to H_2_S**

Morphological divergence between *G. hubbsi* from Archie’s Blue Hole (population 23) and conspecifics from nearby non-sulfidic habitats was weak, and tended to be opposite to our prediction. This may be related to oxygen availability, because efficient oxygen acquisition (not H_2_S per se) is likely the driver for larger heads in sulfidic environments (see discussion and references in Tobler et al. 2008; Crispo and Chapman 2011). Fragmented, shallow tidal creeks (like both populations 25 and 26 investigated here) often experience periods of severe hypoxia (similar to H_2_S-rich environments), in particular during the hot summer months (Lerberg et al. 2000; MacPherson et al. 2007). This could indeed select for even larger heads in mosquitofish from some tidal creeks compared to those from the sulfidic Archie’s Blue Hole (e.g., Crispo and Chapman 2011).

We also uncovered a unique evolutionary pattern in *G. holbrooki* from Green Springs (population 5) where phenotypic divergence was strong but neutral genetic differentiation comparably weak. An educational sign at Green Springs County Park informs the visitor that the sulfidic source for this sinkhole is known to temporarily run dry (R. Riesch, personal observation). Temporal disappearance of H_2_S could lead to occasional influx of non-adapted individuals from nearby Lake Monroe (population 6). Even though these immigrants are likely to suffer from initially high mortality when the habitat turns sulfidic again, interbreeding between immigrants and locally-adapted fish could prevent neutral genetic differentiation, while divergent selection on loci involved in the expression of traits mediating adaptation to H_2_S should still cause phenotypic and genomic differentiation.

*Literature Cited*

Crispo, E., and L. J. Chapman. 2011. Hypoxia drives plastic divergence in cichlid body shape. Evol. Ecol. 25:949–964.

Lerberg, S. B., A. F. Holland, and D. M. Sanger. 2000. Responses of tidal creek macrobenthic communities to the effects of watershed development. Estuaries 23:838–853.

MacPherson, T. A., L. B. Cahoon, and M. A. Mallin. 2007. Water column oxygen demand and sediment oxygen flux: patterns of oxygen depletion in tidal creeks. Hydrobiologia 586:235–248.

Tobler, M., T. J. DeWitt, I. Schlupp, F. J. García de León, R. Herrmann, P. G. D. Feulner, R. Tiedemann and M. Plath. 2008. Toxic hydrogen sulfide and dark caves: Phenotypic and genetic divergence across two abiotic environmental gradients in *Poecilia mexicana*. Evolution 62:2643–2659.

**Table S1.** Life histories (mean ± s.e) of female *Gambusia* spp*.* from sulfide springs and nonsulfidic waters. Female GSI: reproductive allocation. ^a^ Estimated marginal means from clade-specific MANCOVAs with ‘SL’ as a covariate. ^b^ Estimated marginal means from a MANCOVA with ‘SL’ and ‘stage of development’ as covariates. ^c^ Estimated using the intercept and slope from a regression of log-transformed embryo dry weight against stage of development.

| Clade | Site ID | H_2_S | *N* | Mean SL [mm] | Female Lean Weight^a^ [mg] | Female Fat  Content [%] | Fecundity^b^ [# offspring] | Female GSI^a^ [%] | Estimated Embryo Dry Weight at Birth^c^ [mg] | Embryo Fat Content^b^ [%] |
| --- | --- | --- | --- | --- | --- | --- | --- | --- | --- | --- |
| *G. affinis* | (1) | + | 31 | 26.68 ± 0.41 | 139.10 ± 5.55 | 2.73 ± 0.57 | 30.08 ± 3.70 | 15.81 ± 1.49 | 1.94 | 13.90 ± 0.65 |
|  | (2) | – | 44 | 30.61 ± 0.43 | 120.82 ± 3.83 | 4.06 ± 0.31 | 21.36 ± 2.37 | 18.87 ± 1.03 | 1.19 | 14.33 ± 0.42 |
|  | (4) | – | 29 | 36.00 ± 1.04 | 131.24 ± 5.85 | 10.99 ± 0.65 | 55.23 ± 3.87 | 25.03 ± 1.57 | 0.81 | 14.96 ± 0.68 |
|  |  |  |  |  |  |  |  |  |  |  |
| *G. holbrooki* | (5) | + | 33 | 27.92 ± 0.56 | 101.33 ± 2.40 | 8.73 ± 0.93 | 4.57 ± 0.54 | 16.45 ± 0.85 | 5.55 | 17.86 ± 0.58 |
|  | (6) | – | 29 | 26.87 ± 0.58 | 99.31 ± 2.59 | 8.54 ± 0.62 | 9.65 ± 0.58 | 14.60 ± 0.92 | 1.87 | 15.11 ± 0.63 |
|  | (8) | + | 26 | 29.46 ± 0.90 | 107.85 ± 2.72 | 5.59 ± 0.63 | 11.63 ± 0.61 | 21.48 ± 0.97 | 2.50 | 16.12 ± 0.65 |
|  | (9) | – | 31 | 25.72 ± 0.46 | 101.77 ± 2.60 | 4.29 ± 0.53 | 8.39 ± 0.59 | 16.85 ± 0.93 | 1.86 | 15.14 ± 0.64 |
|  | (10) | + | 44 | 29.84 ± 0.38 | 90.35 ± 2.13 | 12.66 ± 0.71 | 12.46 ± 0.48 | 24.80 ± 0.76 | 2.40 | 17.17 ± 0.52 |
|  | (12) | – | 43 | 29.04 ± 0.50 | 92.14 ± 2.11 | 6.37 ± 0.45 | 11.53 ± 0.48 | 21.98 ± 0.75 | 2.43 | 15.86 ± 0.52 |
|  |  |  |  |  |  |  |  |  |  |  |
| *G. eurystoma* / | (13) | + | 14 | 25.29 ± 0.47 | 66.76 ± 2.53 | 6.58 ± 0.64 | 3.02 ± 1.09 | 18.27 ± 1.38 | 5.51 | 16.28 ± 0.46 |
| *G. sexradiata* | (14) | ­– | 30 | 24.17 ± 0.58 | 82.68 ± 1.72 | 10.87 ± 0.81 | 15.79 ± 0.72 | 21.61 ± 0.93 | 1.81 | 17.66 ± 0.69 |
|  |  |  |  |  |  |  |  |  |  |  |
| *G. hubbsi* | (23) | + | 36 | 27.56 ± 0.50 | 66.97 ± 3.15 | 2.35 ± 0.33 | 2.27 ± 0.36 | 11.79 ± 0.57 | 2.67 | 13.95 ± 0.78 |
|  | (25) | ­– | 26 | 24.95 ± 0.77 | 78.01 ± 3.37 | 3.27 ± 0.41 | 5.24 ± 0.38 | 9.65 ± 0.61 | 1.49 | 12.37 ± 0.83 |
|  | (26) | ­– | 11 | 20.31 ± 0.87 | 100.99 ± 6.29 | 4.08 ± 0.53 | 8.48 ± 0.71 | 14.26 ± 1.14 | 1.17 | 8.59 ± 1.54 |

**Table S2.** Life histories (mean ± s.e.) of male *Gambusia* spp*.* from sulfide springs and nonsulfidic waters. GSI: gonadosomatic index. ^a^ Estimated marginal means from a clade-specific MANCOVAs with ‘SL’ as a covariate.

| Clade | Site | H_2_S | *N* | Mean SL [mm] | Male Lean Weight^a^ [mg] | Male Fat  Content [%] | GSI^a^ [%] |
| --- | --- | --- | --- | --- | --- | --- | --- |
| *G. affinis* | (1) | + | 46 | 22.89 ± 0.24 | 37.88 ± 0.76 | 6.28 ± 1.01 | 2.19 ± 0.08 |
|  | (2) | – | 11 | 20.82 ± 0.64 | 45.02 ± 1.47 | 2.37 ± 1.27 | 2.49 ± 0.16 |
|  | (4) | – | 21 | 20.43 ± 0.42 | 39.87 ± 1.13 | 6.89 ± 1.26 | 3.50 ± 0.12 |
|  |  |  |  |  |  |  |  |
| *G. holbrooki* | (5) | + | 23 | 22.67 ± 0.26 | 39.81 ± 0.95 | 7.37 ± 0.88 | 1.98 ± 0.10 |
|  | (6) | – | 20 | 21.34 ± 0.47 | 42.73 ± 1.03 | 6.05 ± 0.84 | 1.66 ± 0.11 |
|  | (8) | + | 13 | 22.61 ± 0.57 | 43.27 ± 1.26 | 4.83 ± 1.21 | 2.02 ± 0.14 |
|  | (9) | – | 18 | 19.21 ± 0.45 | 43.83 ± 1.29 | 3.05 ± 0.57 | 1.95 ± 0.14 |
|  | (10) | + | 24 | 24.43 ± 0.42 | 45.44 ± 1.04 | 12.31 ± 3.62 | 2.38 ± 0.11 |
|  | (12) | – | 24 | 22.34 ± 0.36 | 39.42 ± 0.92 | 5.87 ± 1.09 | 2.18 ± 0.10 |
|  |  |  |  |  |  |  |  |
| *G. eurystoma* / | (13) | + | 27 | 18.22 ± 0.28 | 25.77 ± 0.78 | 4.92 ± 0.75 | 3.18 ± 0.19 |
| *G. sexradiata* | (14) | ­– | 22 | 20.27 ± 0.33 | 35.60 ± 0.88 | 8.58 ± 0.63 | 2.36 ± 0.21 |
|  |  |  |  |  |  |  |  |
| *G. hubbsi* | (23) | + | 20 | 21.53 ± 0.40 | 25.35 ± 1.22 | 2.91 ± 0.34 | 1.21 ± 0.13 |
|  | (25) | ­– | 15 | 18.29 ± 0.41 | 25.96 ± 1.18 | 1.34 ± 0.63 | 1.32 ± 0.12 |
|  | (26) | ­– | 19 | 17.60 ± 0.56 | 34.03 ± 1.15 | 0.12 ± 0.12 | 2.23 ± 0.12 |

**Table S3.** Estimated marginal means (EMM) ± s.e. for the significant clade × H_2_S × sex-effect from the nested mixed-model ANOVA on SL (Table 4a, see main manuscript) and the nested MANCOVA on lean weight, fat content and GSI (at SL = 25.18 mm; Table 4b, see main manuscript).

| Clade | Habitat type | Sex | SL [mm] | Lean Weight [mg] | Fat Content [%] | GSI [%] |
| --- | --- | --- | --- | --- | --- | --- |
| *Gambusia affinis* | non-sulfidic | male | 19.99 ± 0.51 | 86.79 ± 2.71 | 4.56 ± 0.97 | 2.87 ± 0.79 |
|  |  | female | 33.13 ± 0.34 | 69.95 ± 2.24 | 7.64 ± 0.81 | 22.35 ± 0.65 |
|  | sulfidic | male | 22.89 ± 0.42 | 69.13 ± 2.08 | 6.24 ± 0.75 | 2.11 ± 0.61 |
|  |  | female | 26.68 ± 0.51 | 55.75 ± 2.49 | 2.75 ± 0.90 | 15.57 ± 0.73 |
|  |  |  |  |  |  |  |
| *Gambusia holbrooki* | non-sulfidic | male | 20.95 ± 0.36 | 82.25 ± 1.93 | 4.93 ± 0.70 | 1.87 ± 0.56 |
|  |  | female | 27.23 ± 0.28 | 62.35 ± 1.43 | 6.43 ± 0.52 | 17.85 ± 0.42 |
|  | sulfidic | male | 23.35 ± 0.37 | 71.03 ± 1.89 | 8.14 ± 0.68 | 2.01 ± 0.55 |
|  |  | female | 29.03 ± 0.29 | 64.91 ± 1.57 | 9.05 ± 0.57 | 21.05 ± 0.46 |
|  |  |  |  |  |  |  |
| *Gambusia eurystoma/sexradiata* | non-sulfidic | male | 20.27 ± 0.61 | 96.82 ± 3.08 | 8.51 ± 1.11 | 2.12 ± 0.90 |
|  |  | female | 24.17 ± 0.52 | 89.62 ± 2.53 | 10.86 ± 0.91 | 21.81 ± 0.74 |
|  | sulfidic | male | 18.22 ± 0.55 | 99.98 ± 2.96 | 4.82 ± 1.06 | 3.04 ± 0.86 |
|  |  | female | 25.29 ±0 .76 | 75.43 ± 3.69 | 6.58 ± 1.33 | 17.77 ± 1.07 |
|  |  |  |  |  |  |  |
| *Gambusia hubbsi* | non-sulfidic | male | 18.06 ± 0.49 | 105.79 ± 2.74 | 0.62 ± 0.99 | 1.61 ± 0.80 |
|  |  | female | 23.04 ± 0.49 | 84.37 ± 2.53 | 3.63 ± 0.91 | 12.15 ± 0.74 |
|  | sulfidic | male | 21.53 ± 0.64 | 77.51 ± 3.16 | 2.86 ± 1.14 | 1.00 ± 0.92 |
|  |  | female | 27.56 ± 0.48 | 63.37 ± 2.34 | 2.38 ± 0.84 | 11.68 ± 0.68 |

**Table S4.** Results of nested univariate analyses of covariance (ANCOVA) examining life-history and body shape variation of *Gambusia* spp*.* from four different clades based on the divergence vectors derived from the respective MANCOVA. The site(clade × H_2_S)-term was designated a random effect.

| Effect | *F* | df | *P* |
| --- | --- | --- | --- |
| *(a) Adult life histories* | | | |
| SL | 1308.3 | 1, 702 | < 0.0001 |
| Sex | 392.5 | 1, 702 | < 0.0001 |
| Clade | 0.6 | 3, 6 | 0.62 |
| H_2_S | 10.9 | 1, 6 | 0.096 |
| Site(Clade × H_2_S) | 14.8 | 6, 702 | < 0.0001 |
| SL × Sex | 6.2 | 1, 702 | 0.013 |
| SL × Clade | 3.4 | 3, 702 | 0.017 |
| SL × H_2_S | 4.3 | 1, 702 | 0.039 |
| Sex × Clade | 2.0 | 3, 702 | < 0.0001 |
| Sex × H_2_S | 4.3 | 1, 702 | < 0.0001 |
| Clade × H_2_S× Sex | 16.9 | 3, 708 | < 0.0001 |
|  |  |  |  |
| *(b) Offspring-related life histories* | | | |
| SL | 4.4 | 1, 407 | 0.037 |
| Stage | 1.2 | 1, 407 | 0.27 |
| Clade | 5.0 | 3, 10 | 0.022 |
| H_2_S | 16.8 | 1, 10 | 0.002 |
| Site(Clade × H_2_S) | 65.1 | 9, 407 | < 0.0001 |
| Stage × Clade | 3.1 | 3, 407 | 0.026 |
| Stage × H_2_S | 4.6 | 1, 407 | 0.033 |
|  |  |  |  |
| *(c) Body shape* | | | |
| Centroid size | 4.7 | 1, 897 | 0.030 |
| Sex | 1.0 | 1, 897 | 0.31 |
| Clade | 4.0 | 3, 9 | 0.047 |
| H_2_S | 31.2 | 1, 9 | < 0.0001 |
| Site(Clade × H_2_S) | 10.9 | 9, 897 | < 0.0001 |
| Sex × Clade | 17.6 | 3, 897 | < 0.0001 |
| Sex × H_2_S | 5.1 | 1, 897 | 0.025 |
| Clade × H_2_S | 11.2 | 3, 9 | 0.002 |
| Sex × Clade × H_2_S | 4.9 | 3, 897 | 0.002 |

**Table S5.** Descriptive statistics of genetic variability for 11 microsatellite loci across four species and 17 populations of mosquitofish. For each population and locus, observed (*H*_o_) and expected (*H*_E_) heterozygosities as well as allelic richness (*A*) are given. Zero values indicate that the locus is monomorphic in that particular population. Ranges of allele sizes are given for the entire data set.

| Locus | Number of alleles | Range of allele size | Test | (5) G. Springs | (6) Lake Monroe | (7) Flood Drain | (8) P.M.Springs | (9) Ditch Hwy 98 | (10) N. Springs | (11) St. M. River | (12) Ditch St. M. | (13) B.d.Azufre | (16) Pich.2 | (17) Pich.3 | (18) L.W. | (19) P.Lima | (20) C.Villa | (23) Archie's B. H. | (24) St. Creek N. | (26) Th./Scott |
| --- | --- | --- | --- | --- | --- | --- | --- | --- | --- | --- | --- | --- | --- | --- | --- | --- | --- | --- | --- | --- |
|  |  |  |  |  |  |  |  |  |  |  |  |  |  |  |  |  |  |  |  |  |
| Gaaf7 | 27 | 125-249 | *H_O_* | 0.59 | 0.64 | 0.57 | 0.29 | 0.36 | 0.85 | 0.63 | 0.63 | 0.04 | 0.17 | 0.10 | 0.32 | 0.67 | 0.00 | 0.88 | 0.46 | 0.44 |
|  |  |  | *H_E_* | 0.86 | 0.66 | 0.73 | 0.71 | 0.73 | 0.77 | 0.78 | 0.74 | 0.04 | 0.22 | 0.10 | 0.58 | 0.62 | 0.16 | 0.81 | 0.58 | 0.48 |
|  |  |  | *A* | 8.43 | 6.14 | 7.49 | 5.93 | 3.96 | 6.13 | 5.61 | 5.90 | 1.42 | 1.97 | 2.00 | 2.90 | 3.40 | 1.90 | 7.92 | 3.50 | 2.00 |
| Gaaf9 | 14 | 208-268 | *H_O_* | 0.77 | 0.59 | 0.57 | 0.65 | 0.82 | 0.60 | 0.92 | 0.75 | 0.00 | 0.00 | 0.00 | 0.21 | 0.08 | 0.00 | 0.33 | 0.04 | 0.04 |
|  |  |  | *H_E_* | 0.69 | 0.70 | 0.57 | 0.72 | 0.75 | 0.77 | 0.78 | 0.69 | 0.00 | 0.00 | 0.10 | 0.56 | 0.42 | 0.16 | 0.38 | 0.04 | 0.04 |
|  |  |  | *A* | 3.91 | 4.82 | 4.28 | 4.43 | 4.69 | 5.81 | 6.15 | 4.77 | 1.00 | 1.00 | 1.76 | 2.78 | 2.00 | 1.90 | 2.00 | 1.42 | 1.40 |
| Gaaf10 | 66 | 163-427 | *H_O_* | 0.41 | 0.82 | 0.70 | 0.87 | 0.64 | 0.55 | 0.91 | 0.75 | 0.88 | 0.83 | 0.47 | 0.29 | 0.24 | 0.52 | 0.79 | 0.79 | 0.72 |
|  |  |  | *H_E_* | 0.66 | 0.81 | 0.82 | 0.86 | 0.71 | 0.85 | 0.93 | 0.92 | 0.87 | 0.73 | 0.66 | 0.37 | 0.81 | 0.83 | 0.80 | 0.89 | 0.79 |
|  |  |  | *A* | 4.72 | 9.09 | 5.83 | 10.46 | 5.63 | 8.87 | 11.77 | 11.04 | 9.74 | 5.71 | 5.09 | 4.44 | 7.05 | 7.43 | 6.03 | 9.32 | 7.21 |
| Gaaf13 | 44 | 123-371 | *H_O_* | 0.77 | 0.73 | 0.91 | 0.59 | 0.82 | 0.90 | 1.00 | 0.82 | 0.87 | 0.83 | 0.94 | 0.41 | 0.23 | 0.79 | 0.00 | 0.00 | 0.00 |
|  |  |  | *H_E_* | 0.82 | 0.94 | 0.95 | 0.88 | 0.93 | 0.96 | 0.95 | 0.93 | 0.86 | 0.93 | 0.93 | 0.65 | 0.40 | 0.95 | 0.00 | 0.00 | 0.00 |
|  |  |  | *A* | 8.00 | 12.12 | 13.15 | 11.19 | 11.57 | 13.50 | 13.05 | 11.95 | 7.17 | 13.70 | 12.42 | 7.88 | 5.14 | 13.52 | 1.00 | 1.00 | 1.00 |
| Gaaf15 | 12 | 106-154 | *H_O_* | 0.36 | 0.27 | 0.26 | 0.53 | 0.18 | 0.30 | 0.46 | 0.29 | 0.54 | 0.50 | 0.50 | 0.63 | 0.54 | 0.75 | 0.00 | 0.00 | 0.00 |
|  |  |  | *H_E_* | 0.36 | 0.29 | 0.41 | 0.63 | 0.17 | 0.55 | 0.66 | 0.58 | 0.61 | 0.55 | 0.44 | 0.68 | 0.59 | 0.64 | 0.00 | 0.00 | 0.00 |
|  |  |  | *A* | 3.26 | 3.26 | 2.87 | 3.58 | 1.92 | 4.27 | 4.54 | 4.50 | 4.06 | 3.36 | 2.50 | 4.52 | 4.64 | 4.14 | 1.00 | 1.00 | 1.00 |
| Gaaf16 | 25 | 203-355 | *H_O_* | 0.68 | 0.91 | 0.83 | 0.88 | 0.41 | 0.85 | 0.92 | 0.88 | 0.71 | 0.79 | 0.60 | 0.68 | 0.46 | 0.88 | 0.00 | 0.00 | 0.00 |
|  |  |  | *H_E_* | 0.71 | 0.86 | 0.85 | 0.75 | 0.63 | 0.75 | 0.81 | 0.82 | 0.69 | 0.73 | 0.66 | 0.75 | 0.72 | 0.75 | 0.00 | 0.00 | 0.00 |
|  |  |  | *A* | 4.16 | 7.85 | 8.26 | 7.34 | 5.87 | 6.62 | 6.96 | 7.82 | 3.81 | 5.30 | 4.70 | 5.70 | 5.12 | 6.08 | 1.00 | 1.00 | 1.00 |
| Gafu1 | 14 | 90-196 | *H_O_* | 0.59 | 0.73 | 0.83 | 0.41 | 0.61 | 0.40 | 0.33 | 0.23 | 0.00 | 0.22 | 0.07 | 0.69 | 0.14 | 0.21 | 0.00 | 0.25 | 0.00 |
|  |  |  | *H_E_* | 0.62 | 0.74 | 0.72 | 0.52 | 0.60 | 0.43 | 0.38 | 0.33 | 0.16 | 0.20 | 0.19 | 0.52 | 0.48 | 0.34 | 0.00 | 0.33 | 0.00 |
|  |  |  | *A* | 4.79 | 3.99 | 3.91 | 3.90 | 3.48 | 3.72 | 2.48 | 3.40 | 1.90 | 2.34 | 2.56 | 2.77 | 2.73 | 3.54 | 1.00 | 2.78 | 1.00 |
| Gafu2 | 26 | 128-188 | *H_O_* | 0.82 | 0.86 | 0.91 | 0.94 | 0.82 | 0.85 | 0.83 | 1.00 | 0.21 | 0.92 | 0.85 | 0.37 | 0.42 | 0.96 | 0.08 | 0.29 | 0.60 |
|  |  |  | *H_E_* | 0.82 | 0.92 | 0.92 | 0.87 | 0.80 | 0.92 | 0.91 | 0.95 | 0.40 | 0.88 | 0.91 | 0.59 | 0.55 | 0.86 | 0.16 | 0.36 | 0.50 |
|  |  |  | *A* | 7.53 | 10.38 | 10.52 | 7.57 | 5.78 | 11.80 | 10.49 | 12.32 | 3.67 | 9.16 | 9.55 | 6.41 | 5.58 | 8.16 | 1.90 | 2.00 | 2.40 |
| Gafu3 | 41 | 137-291 | *H_O_* | 0.86 | 0.95 | 0.83 | 0.82 | 0.59 | 0.90 | 0.96 | 1.00 | 0.71 | 0.92 | 0.80 | 0.88 | 0.92 | 0.92 | 0.67 | 0.38 | 0.68 |
|  |  |  | *H_E_* | 0.91 | 0.92 | 0.93 | 0.91 | 0.81 | 0.95 | 0.92 | 0.94 | 0.78 | 0.94 | 0.93 | 0.92 | 0.89 | 0.95 | 0.58 | 0.57 | 0.55 |
|  |  |  | *A* | 10.39 | 10.72 | 11.74 | 10.21 | 8.42 | 12.46 | 11.42 | 12.24 | 7.08 | 13.27 | 12.40 | 11.13 | 9.64 | 12.94 | 2.99 | 2.97 | 2.80 |
| Gafu6 | 17 | 185-299 | *H_O_* | 0.64 | 0.77 | 0.87 | 0.59 | 0.52 | 0.95 | 0.79 | 0.58 | 0.00 | 0.42 | 0.30 | 0.11 | 0.54 | 0.25 | 0.00 | 0.08 | 0.00 |
|  |  |  | *H_E_* | 0.82 | 0.91 | 0.89 | 0.68 | 0.61 | 0.86 | 0.88 | 0.82 | 0.08 | 0.43 | 0.50 | 0.73 | 0.77 | 0.64 | 0.00 | 0.08 | 0.00 |
|  |  |  | *A* | 6.46 | 9.33 | 8.60 | 5.11 | 3.95 | 8.18 | 8.76 | 7.64 | 1.67 | 4.52 | 3.76 | 4.79 | 4.91 | 4.97 | 1.00 | 1.67 | 1.00 |
| Gafu7 | 33 | 153-235 | *H_O_* | 0.64 | 0.73 | 0.87 | 0.76 | 0.82 | 0.85 | 0.78 | 0.92 | 0.04 | 0.91 | 1.00 | 1.00 | 0.92 | 0.92 | 0.25 | 0.50 | 0.48 |
|  |  |  | *H_E_* | 0.83 | 0.86 | 0.90 | 0.77 | 0.73 | 0.86 | 0.84 | 0.91 | 0.04 | 0.92 | 0.90 | 0.73 | 0.90 | 0.95 | 0.23 | 0.58 | 0.50 |
|  |  |  | *A* | 7.57 | 7.34 | 9.31 | 5.46 | 5.46 | 8.17 | 8.35 | 10.07 | 1.42 | 11.86 | 10.52 | 6.00 | 10.40 | 13.66 | 2.36 | 2.99 | 2.00 |
|  |  |  |  |  |  |  |  |  |  |  |  |  |  |  |  |  |  |  |  |  |
| Mean across loci |  |  | *H_O_* | 0.65 | 0.73 | 0.74 | 0.67 | 0.60 | 0.73 | 0.77 | 0.71 | 0.36 | 0.59 | 0.51 | 0.51 | 0.47 | 0.56 | 0.27 | 0.25 | 0.27 |
|  |  |  | *H_E_* | 0.74 | 0.78 | 0.79 | 0.76 | 0.68 | 0.79 | 0.80 | 0.79 | 0.41 | 0.59 | 0.58 | 0.64 | 0.65 | 0.66 | 0.27 | 0.31 | 0.26 |
|  |  |  | *A* | 6.29 | 7.73 | 7.81 | 6.83 | 5.52 | 8.14 | 8.14 | 8.33 | 3.90 | 6.56 | 6.11 | 5.39 | 5.51 | 7.11 | 2.56 | 2.69 | 2.07 |

**Table S6.** Offspring-related life histories (mean ± s.e.) of female *Poecilia* spp*.* from sulfide springs and nonsulfidic waters. ^a^ Estimated marginal means from a MANCOVA with ‘SL’ and ‘stage of development’ as covariates. ^b^ Estimated using the intercept and slope from a regression of log-transformed embryo dry weight against stage of development.

| River System | Species | H_2_S | *N* | Mean SL [mm] | Fecundity^a^ [# offspring] | Estimated Embryo Dry Weight at Birth^b^ [mg] | Embryo Fat Content^a^ [%] |
| --- | --- | --- | --- | --- | --- | --- | --- |
| Río Puyacatengo | *P. mexicana* | + | 15 | 30.27 ± 0.64 | 13.53 ± 2.08 | 2.50 | 17.51 ± 1.95 |
|  | *P. mexicana* | – | 31 | 42.68 ± 0.63 | 19.06 ± 1.50 | 2.62 | 19.22 ± 1.41 |
|  |  |  |  |  |  |  |  |
| Río Pichucalco | *P. sulphuraria* | + | 27 | 27.63 ± 0.59 | 12.42 ± 2.14 | 4.53 | 19.87 ± 2.01 |
|  | *P. mexicana* | ­– | 37 | 42.62 ± 0.56 | 15.89 ± 1.42 | 2.77 | 18.92 ± 1.33 |

**Figure S1**. The 13 landmarks for the morphological analyses included (1) the anterodorsal tip of the upper jaw, (2) the anterior and (3) posterior insertions of the dorsal fin, (4) the dorsal and (5) ventral insertions of the caudal fin, (6) the posterior and (7) anterior insertions of the anal fin, (8) the anterior insertion of the pelvic fin, (9) the bottom of the head where the operculum meets the body outline, (10) the dorsal endpoint of the opercular bone, (11) the center of the eye, as well as (12) the dorsal and (13) ventral insertions of the pectoral fin.


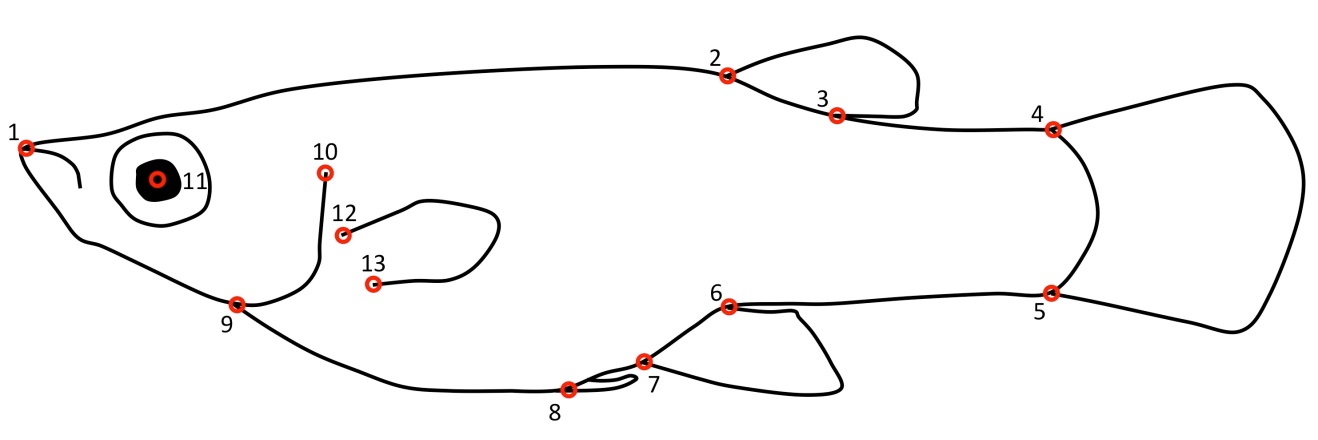


**Figure S2.** Population assignment using STRUCTURE version 2.3.2 for populations of *G. holbrooki* inhabiting sulfidic and surrounding non-sulfidic habitats in the Florida Panhandle. *K* = 2 was recovered as the most likely number of genetic clusters; given are individual relative assignment values in yellow and blue; populations are divided by a black line, and individuals in each population are sorted by the population-specific assignments.


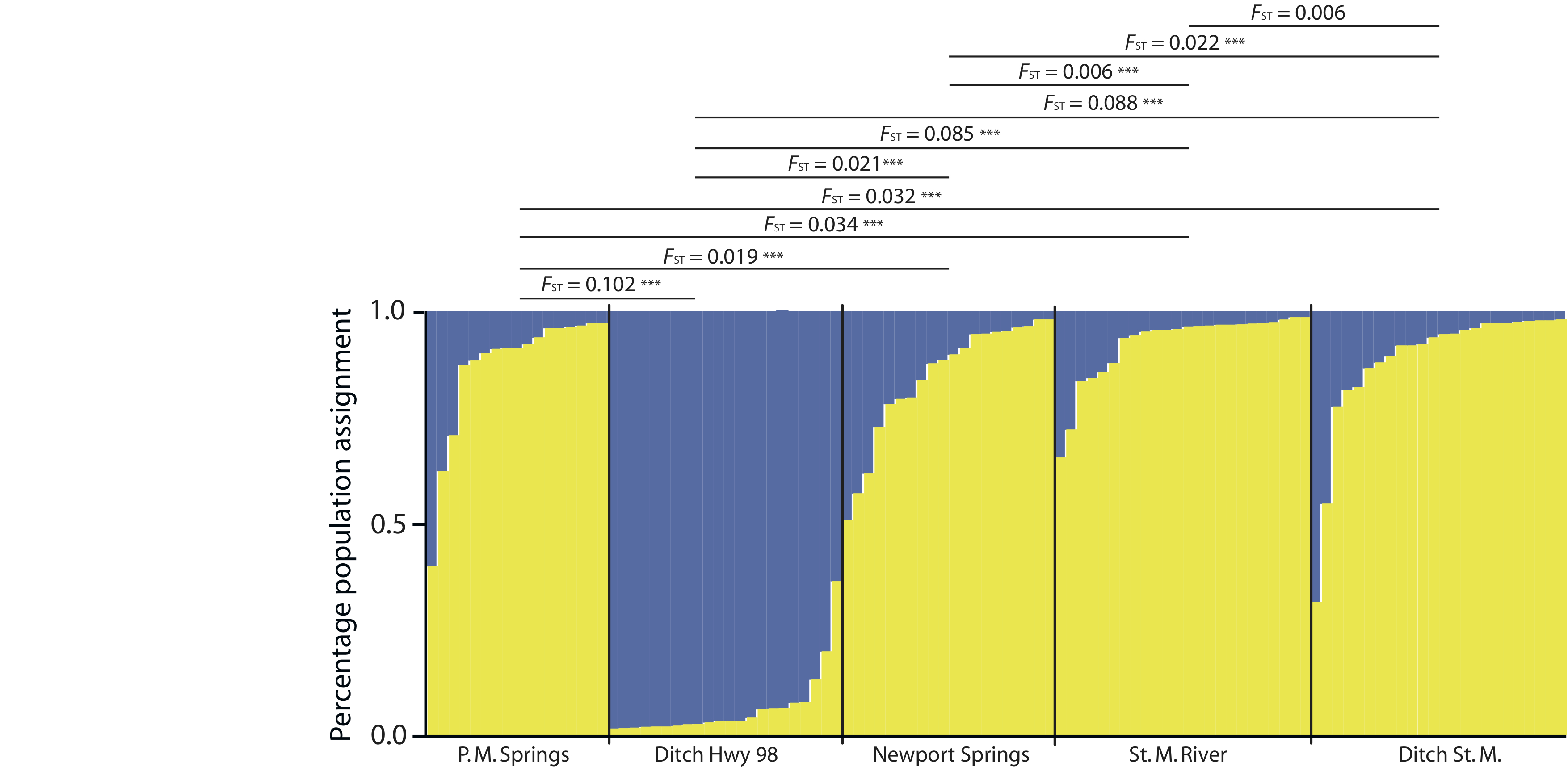


**Figure S3**. Comparison of allelic richness (*A*) and observed heterozygosity (*H*_o_) between *Gambusia* spp. populations inhabiting sulfidic and non-sulfidic habitats. Wilcoxon signed rank test indicated no significant differences (see main text).


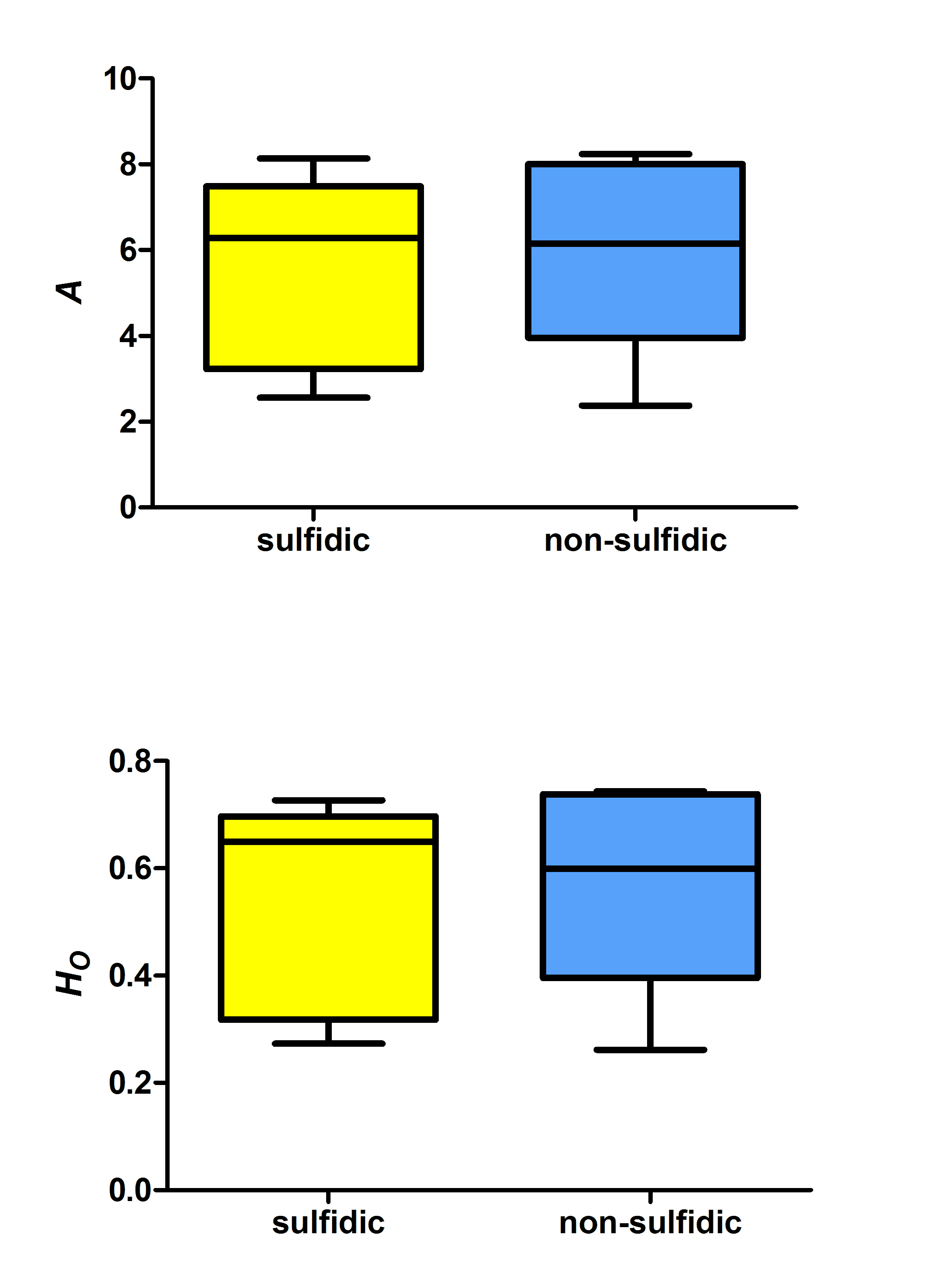


**Figure S4.** First-generation migrants between *Gambusia* spp. from sulfidic and adjacent non-sulfidic sites as calculated by GENECLASS2. Numbers of migrants are given as a proportion of the number of individuals in the recipient population, whereby the direction of the arrows indicates the direction of migration events. Sulfidic and surrounding non-sulfidic habitats of (A-C) *G. holbrooki*, (D) *G. eurystoma/sexradiata*, and (E) *G. hubbsi*. Numbers correspond to sites as described in Table 1.

**
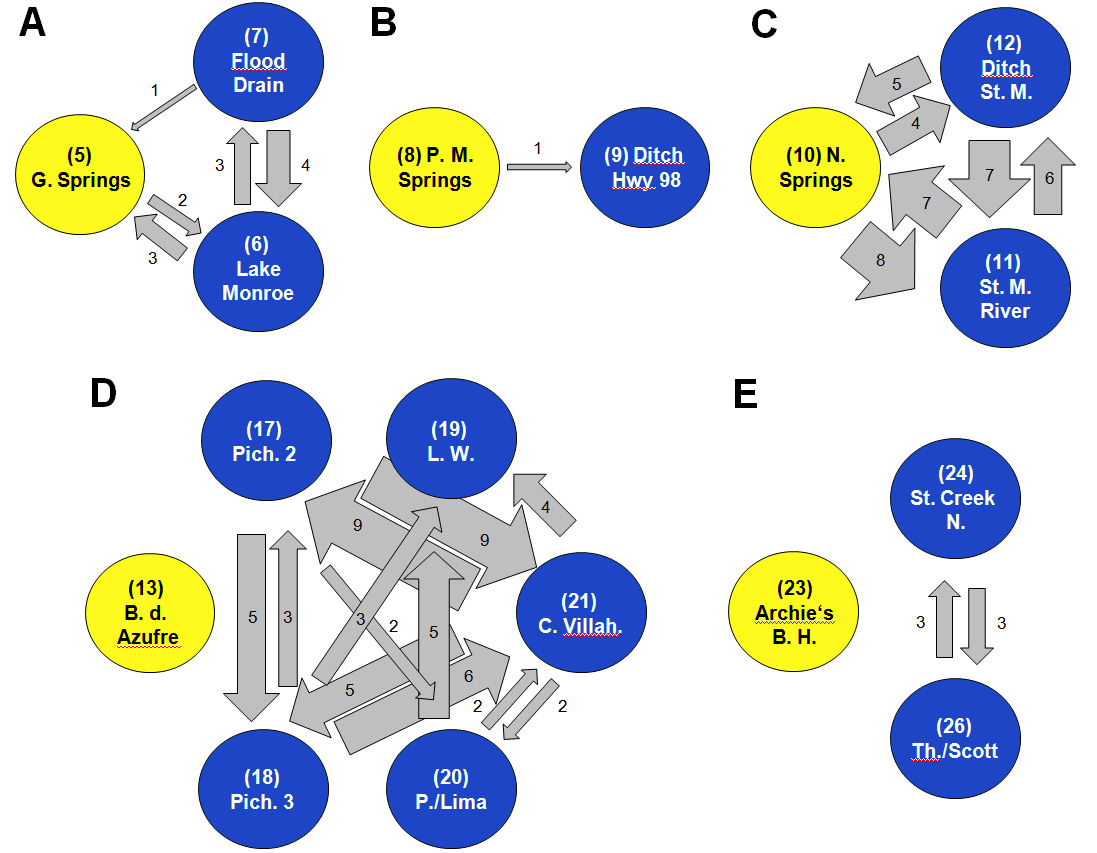
**

**Figure S5.** First-generation migrants in the genus *Poecilia* from sulfidic and adjacent non-sulfidic sites as calculated by GENECLASS2. Numbers of migrants are given as a proportion of the number of individuals in the recipient population, whereby the direction of the arrows indicates the direction of migration events. (A) Sulfidic El Azufre and non-sulfidic Arroyo Bonita; (B) sulfidic La Lluvia and non-sulfidic Río Puyacatengo; (C) sulfidic Baños del Azufre and La Gloria, and non-sulfidic Arroyo Rosita. Numbers correspond to sites as described in Table 1.

**
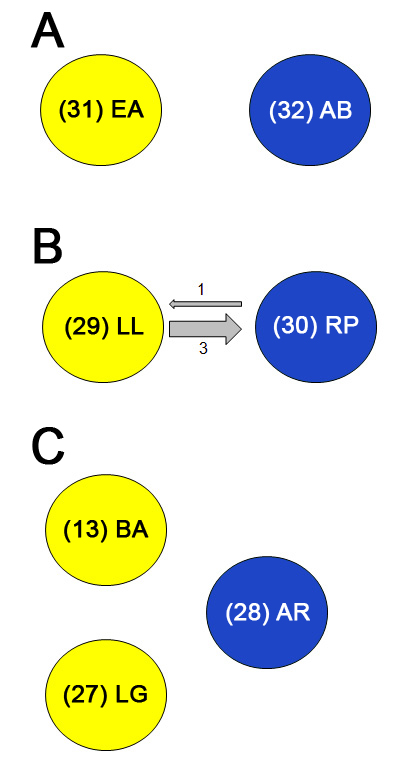
**

**Figure S6.** Relationship between neutral genetic differentiation (*F*_ST_) and (A) mean genetic assignment to the nonresident genetic cluster (essentially a proxy for the proportion of genetic introgression), and (B) numbers of first-generation migrants as calculated by GENECLASS2; see OSM 6 for details. Black circles represent sulfidic systems inhabited by *Gambusia*, grey circles represent sulfidic systems inhabited by *Poecilia*. Regression lines and 95% CI are given in grey.


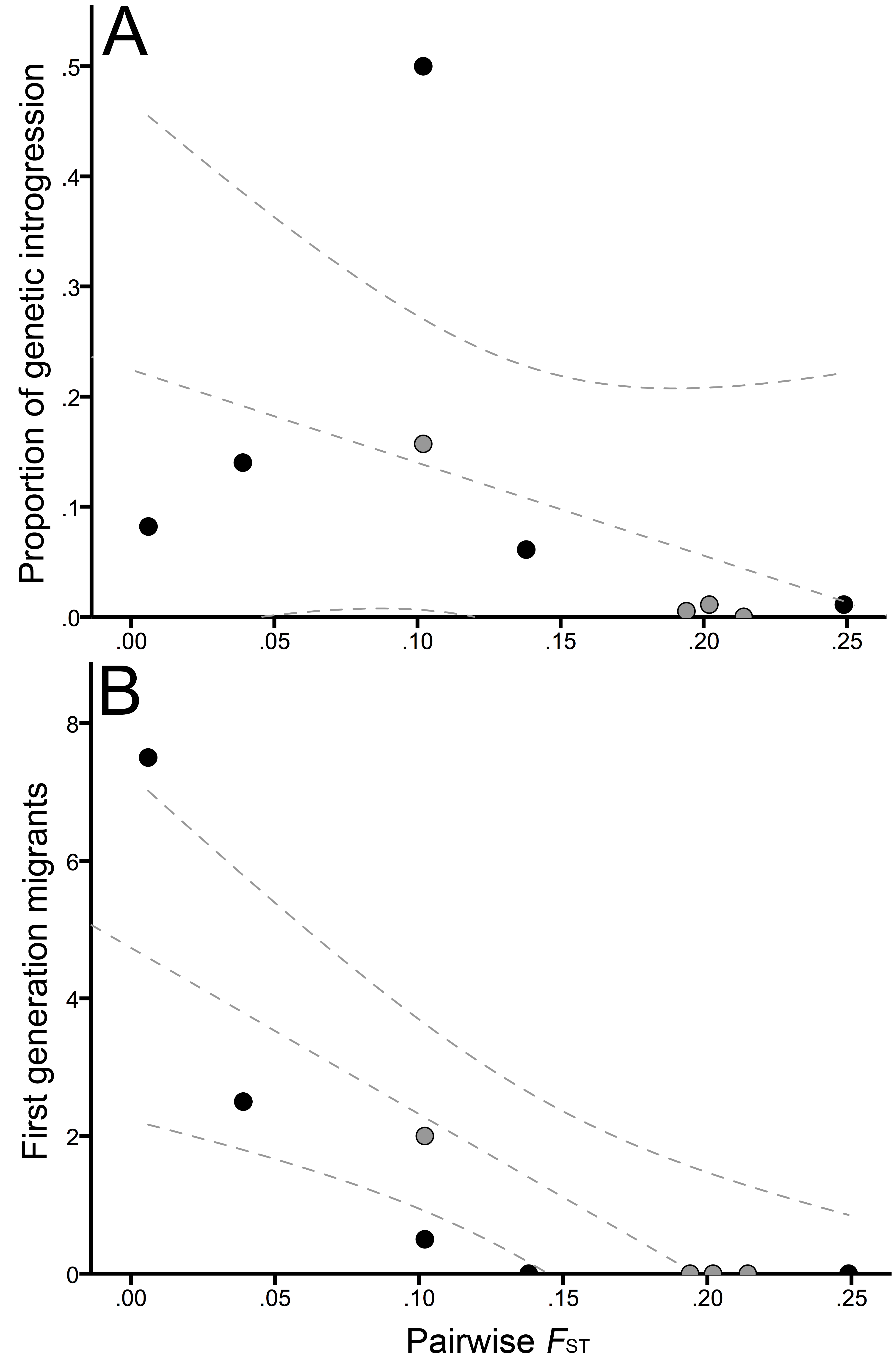

Supplement: Additional file 1: — OSM 1: Additional information on Gambusia collection sites. (DOCX 2179 kb) [file 12862_2016_705_MOESM1_ESM.docx]
